# Oxidative Stress in Amyotrophic Lateral Sclerosis: Pathophysiology and Opportunities for Pharmacological Intervention

**DOI:** 10.1155/2020/5021694

**Published:** 2020-11-15

**Authors:** Teresa Cunha-Oliveira, Liliana Montezinho, Catarina Mendes, Omidreza Firuzi, Luciano Saso, Paulo J. Oliveira, Filomena S. G. Silva

**Affiliations:** ^1^CNC-Center for Neuroscience and Cell Biology, University of Coimbra, UC Biotech Building, Biocant Park, Cantanhede, Portugal; ^2^Center for Investigation Vasco da Gama (CIVG), Escola Universitária Vasco da Gama, Coimbra, Portugal; ^3^Coimbra College of Agriculture, Polytechnic Institute of Coimbra (ESAC, IPC), Bencanta, Coimbra, Portugal; ^4^Medicinal and Natural Products Chemistry Research Center, Shiraz University of Medical Sciences, Shiraz, Iran; ^5^Department of Physiology and Pharmacology “Vittorio Erspamer”, Sapienza University of Rome, Italy

## Abstract

Amyotrophic lateral sclerosis (ALS), also known as Lou Gehrig's disease or Charcot disease, is a fatal neurodegenerative disease that affects motor neurons (MNs) and leads to death within 2–5 years of diagnosis, without any effective therapy available. Although the pathological mechanisms leading to ALS are still unknown, a wealth of evidence indicates that an excessive reactive oxygen species (ROS) production associated with an inefficient antioxidant defense represents an important pathological feature in ALS. Substantial evidence indicates that oxidative stress (OS) is implicated in the loss of MNs and in mitochondrial dysfunction, contributing decisively to neurodegeneration in ALS. Although the modulation of OS represents a promising approach to protect MNs from degeneration, the fact that several antioxidants with beneficial effects in animal models failed to show any therapeutic benefit in patients raises several questions that should be analyzed. Using specific queries for literature search on PubMed, we review here the role of OS-related mechanisms in ALS, including the involvement of altered mitochondrial function with repercussions in neurodegeneration. We also describe antioxidant compounds that have been mostly tested in preclinical and clinical trials of ALS, also describing their respective mechanisms of action. While the description of OS mechanism in the different mutations identified in ALS has as principal objective to clarify the contribution of OS in ALS, the description of positive and negative outcomes for each antioxidant is aimed at paving the way for novel opportunities for intervention. In conclusion, although antioxidant strategies represent a very promising approach to slow the progression of the disease, it is of utmost need to invest on the characterization of OS profiles representative of each subtype of patient, in order to develop personalized therapies, allowing to understand the characteristics of antioxidants that have beneficial effects on different subtypes of patients.

## 1. Introduction

Amyotrophic lateral sclerosis (ALS), also known as Lou Gehrig's disease or Charcot disease, is the most common fatal motor neuron disorder. This neurodegenerative disease is characterized by the progressive loss of upper motor neurons in the cerebral cortex and lower motor neurons in the brain stem and spinal cord, leading to muscle weakness, and progressing into muscle atrophy and paralysis, which culminates in respiratory failure and death [1, 2]. On average, ALS patients have a survival of about 2-3 years from diagnosis, being estimated that only 5-10% of patients survive more than 10 years after diagnosis [3]. So far, no disease-modifying treatment modality has been found for ALS. Currently, there are only two drugs approved by the US Food and Drug Administration for ALS treatment, riluzole, which is a neuroprotective agent that only extends the ALS life expectancy about 3 months, and edaravone, which is an antioxidant that only delays ALS development [4] in some patients [5], as detailed in Section 3.9. This fatal neurodegenerative disease has a worldwide prevalence of 4-6 cases in 100,000 and typically has a late-onset with symptoms arising between 55 and 65 years of age, on average [3]. Generally, men present with an earlier age of onset compared to women, and they are more prone to spinal-onset, whereas bulbar-onset is more frequent in women [6]. The most common form of ALS is sporadic (sALS), with no known etiology, accounting for nearly 90-95% of all the cases, while the remaining 5–10% of the cases are inherited (Familial ALS-fALS), and frequently associated with an earlier age of onset [2, 7].

Although the causes of sALS are still unknown, the disease has been associated with different risk factors, including age, smoking, body mass index, level of physical fitness, and occupational and environmental risk factors, such as exposure to chemicals, pesticides, metals, and electromagnetic fields [8]. However, as none of these external parameters are considered as direct factors triggering the development of this disease, it is believed that there are some individual susceptibility factors that coupled to external exposure to environmental factors lead to the development of ALS [9–11]. Over 50 disease-modifying genes have been described in ALS [12]; mutations in chromosome 9 open reading frame 72 (*C9orf72*) [13, 14], Cu^2+^/Zn^2+^ superoxide dismutase type-1 (*SOD1*) [15–18], TAR DNA-Binding (*TARDBP*) [19], and fused in sarcoma (*FUS*) [20, 21] are among the most prevalent ones. As neither the mutations nor the environmental risk factors completely describe the etiopathogenesis of this disease, a gene-time-environment model has arisen to explain the development of this disease, considering the development of ALS as a multistep process in which genetic background is one of the several triggering factors [10, 22].

Although the precise pathological mechanisms of ALS are still unknown, it is assumed that fALS and sALS share at least some pathological mechanisms, since they present similar clinical pictures [3, 23]. Many molecular mechanisms have been suggested, including glutamate excitotoxicity, altered RNA metabolism, defective axonal transport, protein misfolding and aggregation, endoplasmic reticulum stress, disrupted protein trafficking, oxidative stress (OS), inflammation, and mitochondrial dysfunction [3, 24, 25].

In this review, we provide an update on the role of OS in ALS that accelerates mitochondrial dysfunction and cell damage. Considering that OS decisively contributes to neurodegeneration in ALS, we also describe antioxidant-based therapeutic strategies that have been suggested for ALS management. Several antioxidant agents have failed to show any meaningful therapeutic benefit or were not sufficiently examined. In this regard, we try to sum up the evidence on the positive and negative outcomes for each drug with the aim of achieving novel opportunities for intervention.

## 2. Evidence on the Involvement of Oxidative Stress in ALS

Reactive oxygen species (ROS) are radical or nonradical oxygen species formed by the partial reduction of oxygen, such as superoxide radical anion (O_2_^•-^), hydrogen peroxide (H_2_O_2_), and hydroxyl radical (HO^•^), which are generated as cellular metabolic by-products through enzymatic and nonenzymatic reactions [26]. Mitochondria are one of the most important sites of intracellular ROS production due to their main role in oxidative ATP production, in which molecular oxygen is reduced to water in the electron transport chain [27, 28]. The O_2_^•-^ is produced at a number of sites in mitochondria, including complexes I and III of the electron transport chain [27, 29], pyruvate dehydrogenase [30], and 2-oxoglutarate dehydrogenase [31, 32], all directing ROS towards the mitochondrial matrix (MM), glycerol 3-phosphate dehydrogenase [33] that produces ROS towards the intermembrane mitochondrial space (IMS) [27], and complex III that can leak electrons to oxygen on both sides of the inner mitochondrial membrane (IMM) [34]. Other proteins involved in mitochondrial ROS generation include cytochrome P450 (CYP) enzymes [35], dihydroorotate dehydrogenase [36, 37], complex II [38], and monoamine oxidases [39] which can also contribute to mitochondrial ROS production. Outside mitochondria, several enzymes have also been identified as major sources of ROS, including the nicotinamide adenine dinucleotide phosphate oxidase (NOX), xanthine oxidase, cycloxygenases, CYP450, and lipoxygenases [40]. Under normal conditions, the production and the clearance of ROS are balanced [41]. OS arises when the capability of the organism to maintain the balance is compromised by an excess amount of ROS or by defective antioxidant defense and can be manifested in multiple ways, including modifications of the redox state of critical proteins, and hence of their activity [42, 43]. The cellular antioxidant defense is composed of enzymatic and nonenzymatic antioxidants [44]. Superoxide dismutases, catalase (CAT), glutathione peroxidase (GPx), glutathione reductase (GR), and thioredoxin (Trx) are the major enzymatic antioxidants with an important role in the catalytic removal of ROS, while nonenzymatic antioxidants include low molecular weight compounds, as glutathione (GSH), vitamins A, C, and E, flavonoids, and proteins (e.g., albumin, ceruloplasmin, and metallothionein) [45]. An excessive ROS production associated with an inefficient antioxidant defense represents an important pathological feature in ALS [46].

**Figure 1:**
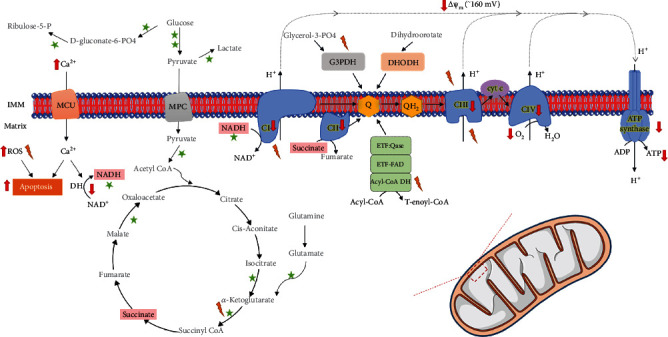
Mitochondrial dysfunction in sporadic forms of ALS. Mitochondrial bioenergetics is driven by the oxidation of different substrates and is stimulated by calcium. Flux of electrons through the electron transport chain creates a transmembrane proton gradient of about 160 mV in the resting state (negative inside), which fuels ATP synthesis in the mitochondrial matrix. Leak of electrons in some of the bioenergetic reactions generates reactive oxygen species (ROS) that are involved in important cellular signaling processes but that, when in excess, may also lead to cellular dysfunction and death. Fibroblasts from sALS patients showed markers of mitochondrial dysfunction, compared to control fibroblasts, including decreased activity of metabolic dehydrogenases, increased ROS levels, increased intracellular calcium levels, decreased expression of components of the oxidative phosphorylation system, decreased mitochondrial potential, oxygen consumption, and ATP levels [68]. Abbreviations: NAD: *β*-Nicotinamide adenine dinucleotide; NADH: *β*-Nicotinamide adenine dinucleotide 2′-phosphate reduced form; FAD: Flavin Adenine Dinucleotide; CI: Complex I; CII: Complex II; CIII: Complex III; CIV: Complex IV; Cyt c: Cytochrome c; ETF: electron transfer flavoprotein; ROS: reactive oxygen species; DH: dehydrogenase; MCU: mitochondrial calcium uniporter; MPC: mitochondrial pyruvate carrier; *ΔΨ*m: mitochondrial transmembrane electric potential; ATP: adenosine triphosphate; ADP: adenosine diphosphate; IMM: inner mitochondrial membrane

A large number of studies have reported increased levels of oxidative damage in proteins, lipids, and DNA of postmortem neuronal tissue [47–49], as well as in cerebrospinal fluid (CSF) [50–53], plasma [54], and urine [55] samples collected from ALS patients, suggesting the involvement of OS mechanisms in the central nervous system (CNS) as well as other tissues. However, it is difficult to determine if oxidative damage represents the primary cause or a secondary consequence of this disease [56, 57] and whether oxidative damage appears early or late in the course of the disease. The impossibility of evaluating OS markers in humans at an early stage of the disease constitutes an obstacle to resolve this riddle, since the initial phase of the disease progresses in a subclinical manner and thus years can pass before the diagnosis. There is no current way to predict which individuals will develop this neurodegenerative disease. On the other hand, the patients' life expectancy is usually very short, and it is not possible to follow OS markers during a long period of time. Nevertheless, animal models can bring some insights. For example, it was described in mutant SOD1 (mutSOD1) mice that the activation of the nuclear factor erythroid-2-related factor 2 (Nrf2)- antioxidant response element (ARE) OS-responsive system occurred in distal muscles before the disease onset [58], supporting the hypothesis that augmented OS in the muscles is implicated in an initial phase of this disease that eventually leads to axonal “dying back” and culminates with motor neurons (MNs) loss. However, it is noted that the studies with animal models that correlate different OS markers with the disease progression only refer to the mutSOD1 model of the disease, which does not represent the majority of patients. For sALS, which represents the highest number of patients, evidence of oxidative damage includes the increase in protein carbonyls [48, 49, 59, 60], 8-hydroxy-2′-deoxyguanosine (8-OHdG) [48, 61], malondialdehyde-modified proteins [48], 4-hydroxynonenal (4-HNE) protein conjugates [61, 62], and nitrotyrosine products [63–65] in spinal cord tissue. Moreover, in erythrocytes from sALS patients, an increase in lipid peroxidation associated with a decrease in CAT, GR, and glucose-6-phosphate dehydrogenase activities and a decrease in GSH, especially in cases with longer disease duration times were measured [66]. The fact that some of the environmental risk factors of ALS, including exposure to agricultural chemicals, heavy metals, excessive physical exertion, chronic head trauma, and smoking, share OS mechanisms as a possible common factor suggests that the appearance of ALS can be facilitated by any factor that favors the prooxidative state [67]. However, the exact oxidative mechanism involved in ALS progression remains to be determined, as well as the real involvement of mitochondria in this process. To clarify this question, Walczak et al. [68] analyzed different parameters of mitochondrial function and antioxidant enzymes to compare sALS patients with fALS patients and controls. Decreased expression of complexes I, II, III, and IV protein subunits was observed in fibroblasts from practically all sALS patients, which also presented lower mitochondrial membrane potential and decreased protein expression of two different antioxidant enzymes: SOD1 and CAT (Figure 1). Principal component analysis allowed a clear separation between 3 classes, corresponding to controls, sALS, and fALS. Controls were mainly characterized by a high expression of SOD1 protein, whereas sALS samples were characterized by high Ci for complexes I and IV (a coefficient that represents the control of metabolic fluxes by a given enzyme), and fALS samples were characterized by a high rate of maximal respiration with substrates for complexes I and II and a high level of the complex I NDUFB8 subunit. These results suggest distinct mechanisms of mitochondrial dysfunction in sALS patients that can lead to chronic mitochondrial stress [68], which should be further clarified in the future.

### 2.1. Association of SOD-1 Mutations with Oxidative Stress in ALS

MutSOD1, accounting for approximately 20% of ALS cases, is one of the most studied causes of ALS, involving OS mechanisms and disruption of mitochondrial function observed in cultured cells [69–71] and in animal models [72–74]. SOD1 is a Cu-Zn metalloprotein responsible for the conversion of O_2_^•-^ into O_2_ and H_2_O_2_ and is localized mainly in the cytosol, being also present in the nucleus, peroxisomes, and mitochondria. This enzyme plays a key role in the antioxidant defense of the cell [75], also regulating cellular respiration and energy metabolism [76]. In ALS patients, there are more than 180 mutations identified across the coding region of the SOD1 gene as well as several others in the noncoding regions [77, 78]. The influence of these mutations on dismutase activity is considerably variable, and they may be associated with a decrease [52], maintenance [79, 80], or increase [52, 81] in the activity compared to wild-type SOD1. Because SOD1 knockout mice do not develop ALS per se [82], and due to the lack of correlation between SOD1 dismutase activity and aggressiveness of clinical phenotypes [83], it has been suggested that mutSOD1 exerts its deleterious effect by a toxic gain of function rather than by altered SOD1 activity [84].

**Figure 2:**
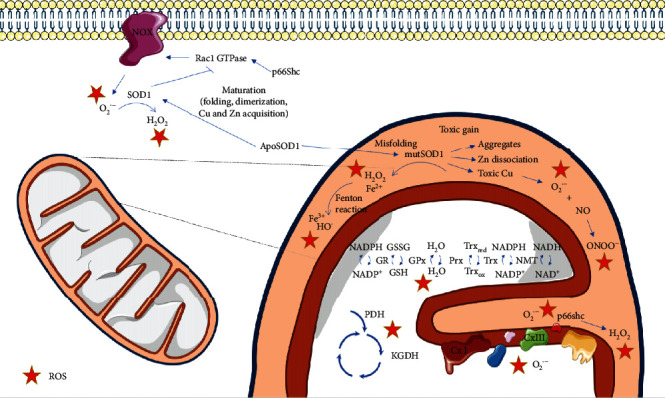
Mitochondrial dysfunction associated with SOD1 mutations. Reactive oxygen species (ROS) may be formed in several cellular reactions and are controlled by a network of antioxidant enzymes that include superoxide dismutase 1 (SOD1), a Cu-Zn metalloprotein responsible for the conversion of O_2_•- into O_2_ and H_2_O_2_, which is mainly localized in the cytosol. SOD1 mutations are one of the most studied causes of ALS. Mutant SOD1 (mutSOD1) toxic gain involves its translocation to the mitochondrial intermembrane space, where it aggregates due to lower stability of mutSOD1 monomers/dimers.mutSOD1 may also cause elevated oxidative damage through the dissociation of zinc from the enzyme or exposure to toxic copper at the active site, promoting reverse O_2_•- production. O_2_•- reacts with nitric oxide generated by nitric oxide synthase, more rapidly than it does with SOD1, producing peroxynitrite, with consequent tyrosine nitration of cellular proteins. mutSOD1 may also act as a peroxidase by using H_2_O_2_ as a substrate, or the H_2_O_2_ produced in the dismutation reaction may originate HO^•^ through the Fenton reaction. mutSOD1 may also induce the activation of p66Shc, a protein involved in controlling mitochondrial redox homeostasis. Outside mitochondria, mutSOD1 associates more strongly with Rac1 compared to the wild type form of SOD1, being less sensitive to redox uncoupling, consequently leading to an increase in NADPH oxidase- (NOX-) derived O_2_•-. ApoSOD1: metal-deficient Cu,Zn-superoxide dismutase; NADP: *β*-Nicotinamide adenine dinucleotide 2′-phosphate; NADPH: *β*-Nicotinamide adenine dinucleotide 2′-phosphate reduced form; NAD: *β*-Nicotinamide adenine dinucleotide; NADH: *β*-Nicotinamide adenine dinucleotide 2′-phosphate reduced form; GSH: reduced glutathione; GSSG: oxidized glutathione; Trxred: reduced Thioredoxin; Trxox: oxidized Thioredoxin; Trx: Thioredoxin, NMT: N-myristoyltransferase; Prx: peroxiredoxin; GPx: glutathione peroxidase; GR: glutathione reductase; PDH: pyruvate dehydrogenase; KGDH: alpha-ketoglutarate dehydrogenase; CxI: complex I; CxIII: complex III.

The mechanism of this toxic gain is currently unknown. A number of hypotheses regarding this toxic property have been proposed, none of them being proven so far: (1) mutSOD1 could act as a peroxidase by using as a substrate the H_2_O_2_ produced through ordinary dismutase reaction [80, 85]; (2) mutSOD1 could react with peroxynitrite to cause tyrosine nitration [42, 86]; and (3) formation of aggregates due to a decrease in the stability of SOD1 monomer/dimers [87]. As a peroxidase, it has been proposed that SOD1 catalyzes the reverse of its normal dismutase reaction or uses the H_2_O_2_ produced in the dismutation as a substrate to produce HO^•^ through the Fenton reaction [88, 89]. It has also been suggested that mutSOD1 causes elevated oxidative damage through the dissociation of zinc from the enzyme [90] or exposure to toxic copper at the active site, promoting reverse O_2_^•-^ production [91]. On the other hand, O_2_^•-^ also reacts with nitric oxide which is generated by nitric oxide synthase, more rapidly than it does with native SOD1, producing peroxynitrite, with consequent tyrosine nitration of cellular proteins [42, 86] (Figure 2).

Notwithstanding, it has also been proposed that the maturation of SOD1 is a complex multistep process, which easily predisposes SOD1 to misfolding or/and polymerization and aggregation [92–94]. In fact, the SOD1 enzyme can itself be a target for OS, leading to possible folding and aggregation defects [95], which remains controversial in ALS pathogenesis. While a correlation was found between the accumulation of SOD1 aggregates and the disease progression in cervical, thoracic, and lumbar spinal cord tissues of B6-SJL-Tg (SOD1^G93A^) mice, it was also suggested that an enhanced capacity of drawing the misfolded SOD1 into aggregates may confer resistance against its own toxicity [96]. Similarly, Zhu et al. [92] showed that low molecular weight nonnative SOD1 trimers were cytotoxic in neuroblastoma cells, while SOD1 aggregates did not affect cell viability. Together, these studies suggest that misfolded SOD1 can be a disease driver, especially for the spinal cord, while SOD1 aggregates are considered benign or protective agents against the disease progression. Indeed, misfolded SOD1 identified in spinal cord mitochondria from both SOD1^G93A^ rats and SOD1^G37R^ mice was associated with an increased susceptibility to OS and mitochondrial damage [72]. Moreover, in the mouse motoneuronal NSC-34 cell line, the mutSOD1 proteins were found to associate with mitochondria due to the oxidation of cysteine residues, which causes mutSOD1 to accumulate in an oxidized, aggregated state. Consequently, the presence of mutSOD1 leads to the impairment of the respiratory chain and a shift in the mitochondrial redox balance (GSH/GSSG ratio) towards a higher level of OS [69] (Table 1). Similarly, Liying et al. [70] reported reduced levels of GSH and enhanced levels of GSSG in NSC34 motor neuron-like cells and lumbar tissues of the spinal cord of mutant SOD^G93A^ mice, suggesting that the decrease in GSH and a higher oxidative state in cells promote apoptotic cell death that contributes, at least partially, to motor neuron degeneration in ALS. Additionally, it was also found that the expression of mutSOD1 in SH-SY5Y human neuroblastoma cells induces the activation of p66Shc, a protein involved in controlling mitochondrial redox homeostasis in neuronal-like cells [71].

The overexpression of mitochondria-targeted CAT improved mitochondrial antioxidant defenses and mitochondrial function in SOD1^G93A^ astrocyte primary cultures, however SOD1^G93A^ mice treated with this antioxidant did not develop the disease later or survive longer, suggesting that preventing peroxide-mediated mitochondrial damage alone is not sufficient to delay the disease [97]. In mutSOD1 ALS models (H46R/G93A rats and G1H/G1L-G93A mice), certain residual motor neurons showed the overexpression of peroxiredoxin-l and glutathione peroxidase-l (Prxl/GPxl) during their clinical courses, while at the terminal stage of ALS, a disruption of this common Prxl/GPxl-overexpression mechanism was observed in neurons, suggesting that the breakdown of this redox system at the advanced disease stage probably accelerates neuronal degeneration and neuronal death [98] (Table 1). Indeed, decreased GSH levels caused motor neuron degeneration in the SOD1^wt^ mice model [99] and accelerated motor neuron death in SOD1^G93A^ mice, by aggravating mitochondrial pathology [73].

Protein cysteine residues are crucial in the regulation of cellular redox balance, due to their thiol groups that can form covalent disulfide bonds, which are critical for correct protein folding, function, and stability [100]. The tripeptide GSH, which contains cysteine, is the major thiol antioxidant and can act as an electron donor to reduce disulfide bonds in proteins. Cysteine thiols are critical for several cellular functions, including signal transduction and DNA binding of redox-responsive transcription factors, such as Nrf2 and nuclear factor kappa-light-chain-enhancer of activated B cells (NF-kB) [101]. SOD1 has four cysteine residues (Cys 5, 57, 111, and 146), and its oligomerization may involve covalent disulfide cross-linking mediated by Cys 111, which is relatively exposed on the protein surface [102], with Cys 6 also playing a possible role [103]. However, this cannot completely explain SOD1 aggregation in ALS because all SOD1 cysteine residues have been found to be mutated in ALS and, therefore, are not present in some patients that present SOD1 aggregates [104].

SOD1 has a tight connection with the Nrf2 pathway, which is a major regulator of the phase II antioxidant response and respective antioxidant elements, including GPx, CAT, GR, and enzymes involved in GSH synthesis and nicotinamide adenine dinucleotide phosphate (NADPH)- regenerating enzymes [105–107]. Nrf2 usually resides in the cytosol bound to Keap1 (Kelch ECH-associating protein 1; the cytoplasmic Nrf2 regulator). Oxidative modification of cysteine residues on Keap1 leads to the release of Nrf2, which in turn translocates to the nucleus upregulating the expression of genes with an ARE in their promoter [108, 109]. Nrf2 expression was found to be decreased in NSC-34 cells expressing mutSOD1, in MNs isolated from familial SOD1-associated ALS patients [110], and in primary motor cortex and spinal cord postmortem tissue samples from ALS patients [111], which suggested that increasing neuronal Nrf2 activity may represent a novel therapeutic target. The endogenous activation of the Nrf2-ARE system during the development of pathology in the SOD1^G93A^ mouse model of ALS showed that the early Nrf2-ARE activation occurs in muscle tissue and that eventually, it progresses in a retrograde manner leading to MN loss [58], as previously described. However, the fact that Nrf2-ARE activation may occur in sALS patients, as well as in those carrying mutSOD1, led these authors to speculate that this pathway is probably independent of mutSOD1 [58].

Nicotinamide adenine dinucleotide phosphate oxidase-dependent redox stress is another mechanism described to be related to mutSOD1. In fact, it has been demonstrated that the deletion of NOX2, and to a lesser extent NOX1, in SOD1^G93A^ transgenic mice, slows down disease progression and improves survival [112, 113]. Accordingly, it was also presumed that SOD1 can regulate NOX2-dependent O_2_^•-^ production by binding to Rac1, also known as Ras-related C3 botulinum toxin substrate 1, leading to the inhibition of its GTPase activity [114]. These authors suggested that in physiological conditions, SOD1 efficiently binds to Rac-GTP and inhibits its GTPase activity, increasing NOX2 activity in reducing conditions, whereas the accumulation of H_2_O_2_ leads to the dissociation of SOD1 from Rac-GTP, promoting the inactivation of Rac through GTP hydrolysis, with consequent NOX2 inactivation and decrease in ROS production. In ALS, mutSOD1 associates more strongly with Rac1 compared to the wild type form of SOD1 (SOD1^G93A^ vs. SOD1^WT^ transgenic mice), being less sensitive to redox uncoupling, consequently leading to the hyperactivation of NOX-derived O_2_^•-^ by endomembranes [114] (Figure 2).

### 2.2. Association of TDP-43 Mutations with Oxidative Stress in ALS

Other less characterized mutated genes linked to ALS have also been associated with OS mechanisms. Mutant TAR DNA-Binding Protein 43 (TDP-43), which has several interactions with the members of the family of heterogeneous nuclear ribonucleoproteins (hnRNPs), has also been reported to affect the Nrf2 pathway [115–117]. Supporting this idea, Moujalled et al. [116] suggested an association between the TDP-43 protein and Nrf2, mediated by the third partner hnRNP K. The same authors showed that fibroblasts from TDP-43^M337V^ patients and astrocyte cultures from TDP-43^Q331K^ mice both displayed impaired levels of GSH (downstream Nrf2 antioxidant), indicating an increase in OS dependent on a disruption of the Nrf2 pathway. The idea of an impairment in the Nrf2/ARE pathway has also been evidenced in studies with TDP43 mutations in NSC-34 cells [115, 117]. NSC-34 cells overexpressing TDP-43^M337V^ showed increased values of intracellular lipid peroxidation, lower cell viability, nuclear accumulation of Nrf2, and decreased protein expression of NAD(P)H quinone dehydrogenase 1 (NQO1, downstream Nrf2 antioxidant), suggesting that TDP-43^M337V^ weakened cellular antioxidant defenses, which turned the cells more susceptible to the increase of OS [115]. Similar results were also described by Duan et al. [117] in NSC-34 cells overexpressing TDP-43^M337V/Q331K^ that showed nuclear accumulation of Nrf2, as well as decreased heme oxygenase (HO-1) protein levels, which is also a phase II detoxification enzyme regulated by the Nrf2 pathway (Table 1).

Similarly to SOD1, cysteine residues are candidates for the mediation of TDP-43 aggregation, although the mechanisms are still not completely explained [118]. TDP-43 has six cysteine residues, four located in RNA recognition motifs (Cys 173, 175, 198, and 244) and two in the N-terminal domain (Cys 39 and 50) [119], with no mutations found so far in ALS [104]. In fact, oxidation of cysteine residues in the RNA recognition motifs was shown to decrease protein solubility and lead to the formation of intra- and intermolecular disulfide bridges [120, 121].

### 2.3. Association of FUS Mutations with Oxidative Stress in ALS

Fus, a hnRNP (hnRNP P2) [122], is involved in DNA damage response induced by DNA-double strand breaks [123, 124], among other pathways, although its role has not been completely clarified. Wang et al. [124] showed that the loss of nuclear FUS in fibroblasts obtained from fALS patients with the R521H and P525L FUS mutations, and in induced pluripotent stem cells (iPSCs)/MNs derived from these fibroblasts, caused the accumulation of unrepaired DNA strand breaks, which culminated in an increased vulnerability to OS, suggesting a protective effect of FUS against OS [124].

### 2.4. Association of C9orf72 Mutations with Oxidative Stress in ALS

Concerning C9orf72, the most prevalent mutation in ALS, few studies have related this mutation with OS mechanisms. In C9orf72-related ALS, the expansion of GGGGCC (G4C2) hexanucleotide is found repeated in the first intron of the C9orf72 gene at least thirty times [125]. The expression of expanded G4C2 repeats results in the production of 5 dipeptide repeat (DPR) proteins: poly-glycine-alanine (poly-GA), poly-glycine-proline (poly-GP), poly-glycine-arginine (poly-GR), poly-proline-alanine (poly-PA), and poly-proline-arginine (poly-PR), which still have an unknown role in ALS progression and OS mechanisms [126, 127]. C9orf72 motor neurons derived from iPSC presented an overexpression of the poly-GR protein and DNA damage that increased gradually with the time of cell culture, possibly due to poly-GR-induced OS [128]. Additionally, these authors reported that poly-GR preferentially binds to mitochondrial ribosomal proteins, compromising mitochondrial function by increasing mitochondrial membrane potential and ROS production, revealing the importance of mitochondrial OS mechanisms in C9orf72-related ALS [128]. Another study with astrocytes derived from mutant C9orf72 iPSC also reported a reduced secretion of several antioxidant proteins by astrocytes, and wild type MNs exposed to media conditioned by these C9orf72-astrocytes showed increased OS [129], suggesting that dysfunction of C9orf72-astrocytes also leads to OS in MNs, contributing to neurodegeneration (Table 1).

### 2.5. Association of Other Less Frequent Mutations with Oxidative Stress in ALS

Mutations in angiogenin (ANG) may occur in 1-2% of fALS patients [130], and there is evidence that it may be involved in OS associated with ALS [67, 131, 132] (Table 1). ANG is a secreted ribonuclease that can cleave some tRNAs and modulate protein translation in neurons. A study in murine astrocytes has shown that ANG activates the Nrf-2 pathway in these cells, and the conditioned medium of these astrocytes protects neuronal cells against H_2_O_2_-induced oxidative damage [133].

Paraoxonases (PONs including PON1, PON-2, and PON-3) are enzymes involved in the neutralization of highly toxic organophosphates, and their polymorphisms have been reported in ALS patients [134–136]. Their antioxidant role has been well studied in cardiovascular diseases [137]; however, PON genetic alterations may also be associated with OS in ALS, especially in the context of organophosphate poisoning, which is one of the well-established ALS risk factors [67].

## 3. Preclinical and Clinical Studies with Antioxidants

Although evidence of oxidative damage in ALS pathogenesis has been largely described in the literature, all antioxidants tested in patients have so far failed, remaining unclear whether any antioxidant therapies might be effective for treating ALS. In this section, we describe various preclinical and clinical trials with antioxidants that have already been completed or are ongoing.

### 3.1. Vitamin E

**Figure 3:**
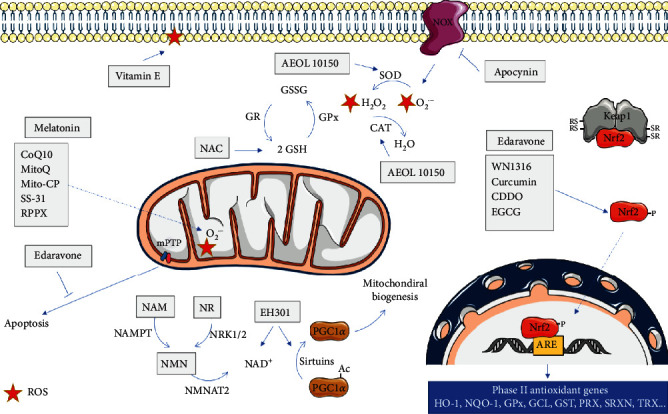
Mitochondrial effects of different antioxidant agents in ALS. The scheme represents the main molecular targets of antioxidants used in ALS, as discussed in the main text. HO-1: heme oxygenase 1; NQO-1: NADPH quinine oxidoreductase 1; GPx: glutathione peroxidase; GCL: *γ*-glutamylcysteine synthetase; GST: glutathione S-transferase; PRX: peroxiredoxin; SRXN: sulfiredoxin; TRX: Thioredoxin; GR: glutathione reductase; CAT: catalase; SOD: superoxide dismutase; NAMPT: nicotinamide phosphoribosyltransferase; NMNAT2: nicotinamide/nicotinic acid mononucleotide adenylyltransferase 2; NRK1/2: nicotinamide riboside kinase 1/2; CoQ10: coenzyme Q10; RPPX: dexpramipexole; NAC:N-acetyl cysteine; CDDO: 2-cyano-3,12-dioxooleana-1,9,-dien-28-oic acid; EGCG: epigallocatechin gallate; ROS: reactive oxygen species.

Vitamin E (alpha-tocopherol) is the most active natural lipophilic antioxidant that protects cell membranes from lipid peroxidation [138, 139] and has been extensively tested in the context of ALS (Figure 3). A preclinical study in SOD1^G93A^ transgenic mice showed that dietary supplementation with vitamin E (200 UI/kg) slowed the disease progression and delayed the onset, but did not affect the survival time [140] (Table 2). Although vitamin E deficiency is not consistently present in ALS patients [141–143], a reduced risk for ALS was described in patients with higher vitamin E levels [141], or in those with low baseline vitamin E levels who were supplemented with vitamin E [144–146]. Despite these positive results, three double-blind, placebo-controlled, clinical trials on ALS patients using oral administration of vitamin E (in a range from 500 mg twice a day to 5000 mg/day) until 18 months of treatment did not affect the quality of life neither the survival of the patients, although ALS progression was slowed [147–149] (Table 2). Although vitamin E did not appear to affect the survival in ALS, patients receiving riluzole plus alpha-tocopherol remained longer in the milder states of ALS, and after 3 months of treatment, they presented an increase in plasma GSH levels and a decrease in plasma thiobarbituric acid reactive species levels [147]. The negative results in human studies may be justified in part by the effect that vitamin E does not readily penetrate the blood-brain barrier (BBB) and does not reach the CNS in sufficient concentration to be efficient. In fact, the mean ventricular CSF concentration of vitamin E was 0.114 *μ*M after an increased monthly dosage (400, 800, 1,600, 3,200, and 4,000 IU/day) over 5 months [150], while its IC_50_ (concentration at which a 50% inhibitory effect is observed) in a variety of *in vitro* radical scavenging assays was between 1.5 and 59 *μ*M [151].

Based on the assumption that supplementation with vitamin E may reduce the risk of ALS and moderately slow ALS progression, a randomized crossover clinical trial in phase III to test the effect of vitamin E on treatment of muscular cramps in ALS patients was initiated in 2006 (NCT00372879); however, the results have not yet been published. A pilot randomized, double-blind, placebo-controlled clinical trial in phase II (NCT04140136) was also initiated in 2019 to investigate the effects of vitamin E mixed tocotrienols in patients with ALS, particularly in delaying disease progression, as well as to assess its safety profile in this group of patients. A Cochrane systematic review found that the evidences on the beneficial effect of vitamin E and other treatment strategies on muscle cramps were not conclusive to support the use of these agents in ALS patients [152].

### 3.2. N-Acetyl-L-Cysteine (NAC)

N-acetyl-L-cysteine (NAC) is a membrane-permeable antioxidant molecule that alleviates free radical damage [153] and replenishes the plasma levels of cysteine, as well as the depleted GSH pools (Figure 3), when administered orally [154]. A preclinical study showed that NAC (1 mM and 24 h) lowered mitochondrial ROS production, returned MTT reduction rate to control levels, and also increased ATP levels in human neuroblastoma SH-SY5Y cell lines carrying G93A SOD1 mutation [155]. Additionally, the administration of NAC (2.0 mg/Kg/day) in SOD1^G93A^ transgenic mice significantly extended survival and improved motor performance [153]. However, in a double-blind placebo-controlled clinical trial on 110 ALS patients, a subcutaneous infusion of NAC (50 mg/kg daily) did not result in a major increase in a 12-month survival or in a reduction of disease progression [156] (Table 2); therefore, the beneficial effects of NAC in ALS remain questionable.

### 3.3. Coenzyme Q10

Coenzyme Q10 (CoQ10), also known as ubiquinone, is a lipophilic antioxidant, as well as an essential mitochondrial cofactor that mediates electron transfer in the respiratory chain [157, 158]. It has been described that CoQ10 exerts beneficial effects in ALS by scavenging free radicals, protecting against OS (Figure 3). The administration of CoQ10 (200 mg/kg daily) significantly increased the mitochondrial concentrations of coenzyme Q10 in the cerebral cortex and prolonged the survival of SOD1^G93A^ transgenic mice when the administration started at 50 days after birth [159]. However, another study showed that the administration of CoQ10 (800 mg/kg/day orally) was unable to prolong the survival of SOD1^G93A^ mice when it started from the onset of disease until death [160]. Controversial results were also found for the serum or plasma CoQ10 concentrations in ALS patients (Table 2). While an increase in the oxidized form of CoQ10 was found in 20 sALS patients compared to controls [161], another study described similar serum concentrations of CoQ10 in 30 ALS patients and controls [162]. CoQ10 has subsequently been shown to be well-tolerated in 31 ALS patients at doses up to 3000 mg/day for 8 months [163]. However, a phase II randomized, placebo-controlled, double-blind, multicenter clinical trial (NCT00243932) with the administration of CoQ10 (2700 mg/day) in ALS patients concluded that the difference between the CoQ10 group and the placebo group was not large enough to justify continuing to a phase III trial [164, 165] (Table 2). The limited pharmacological effect of CoQ10 could be justified by its poor CNS availability after an oral administration [160].

### 3.4. Nrf2/ARE Modulators

The protective role of Nrf2 against neurodegenerative diseases is well described in the literature and may represent a therapeutic target for ALS and other neurological disorders [166]. In fact, the overexpression of Nrf2 in astrocytes in coculture protects motor neurons from SOD1^G93A^ toxicity, increasing the amount of GSH secreted by astrocytes [167]. Crossing SOD1^G93A^ mice with mice overexpressing Nrf2 selectively in astrocytes significantly delayed disease onset and extended survival of SOD1^G93A^ transgenic mice [167], making Nrf2 a possible therapeutic target in ALS. However, contrary to what was expected, Guo et al. [168] reported a slight impact of the Nrf2 knockout on the course of disease in SOD1^G93A^ mice. These authors also demonstrated that the elimination of Nrf2 only affected NQO1, among different Nrf2-regulated phase II enzymes, leaving it an open question whether Nrf2-mediated neuroprotection is a key mechanism to prevent ALS neurodegeneration [168].

Pharmacological targeting of Nrf2/ARE pathways has been proposed as a therapeutic strategy against neurodegenerative disorders, including ALS, since it helps neuronal cells to cope with OS [169]. One example is the case of the novel acylaminoimidazole derivative, 2-[mesityl(methyl)amino]-N-[4-(pyridin-2-yl)-1H-imidazol-2-yl] acetamide trihydrochloride (WN1316) that proved to upregulate Nrf2 and regulate GSH, protecting motor neurons against OS [170] (Figure 3). The oral administration of WN1316 (1-100 *μ*g/kg/day) improved mice motor function and extended the survival of SOD1^H46R^ and SOD1^G93A^ mice [170] (Table 2). Additionally, transgenic mice treated with WN1316 showed reduced oxidative damage to neuronal cells and preserved integrity of the skeletal muscle together with the suppression of astrocytosis and microgliosis in the spinal cord [170]. Although the molecular mechanism of WN1316 is not yet completely understood, the activation of the Nrf2 signaling pathway is thought to take part in this process. Phase I clinical trials of WN1316 (UMIN000015054) were completed in early 2015, but results were not published so far (https://upload.umin.ac.jp/cgi-open-bin/ctr_e/ctr_view.cgi?recptno=R000017516; accessed on 23 July 2020).

Curcumin, a natural and liposoluble dye obtained from turmeric is another compound that modulates the Nrf2 pathway [166] (Figure 3). Curcumin was shown to activate the Nrf2 pathway in primary spinal cord astrocytes, attenuating oxidative damage and mitochondrial dysfunction [171]. Additionally, to these beneficial effects, curcumin was also shown to bind to the prefibrillar aggregates of SOD1 and alter their amyloidogenic pathway, alleviating cytotoxicity [172]. Dimethoxy curcumin improved mitochondrial dysfunction in NSC-34 cell line transfected with human M337V or Q331K mutant TDP-43, suggesting that this compound can be useful to treat neurodegenerative diseases linked with mutated TDP-43 [173]. The oral administration of 80 mg/day nanocurcumin (SinaCurcumin) in a pilot randomized clinical trial using 54 sALS patients during 12 months showed a general improvement in the survival of ALS patients, especially those with bulbar involvement (https://en.irct.ir/trial/11697) [174]. Moreover, in a double-blind clinical trial, curcumin oral supplementation (600 mg/day, Brainoil) in 42 ALS patients during 6 months resulted in a decrease in ALS progression, improvement of aerobic metabolism, and a reduction of oxidative damage [175] (Table 2). Despite these beneficial effects, curcumin chemical instability, low oral bioavailability, and low water solubility constitute an obstacle that has to be overcome during the development of drug delivery systems based on this compound [176, 177].

Adding to the list of Nrf2 modulators, two triterpenoids, CDDO (2-cyano-3, 12-dioxooleana-1,9-dien-28-oic acid) ethylamide (CDDO-EA) and CDDO-trifluoroethylamide (CDDO-TFEA), were also described to activate Nrf2/ARE in SOD1^G93A^ mouse model as well as in a cell culture model of ALS [178]. The treatment of NSC-34 cells with CDDO-TFEA upregulated Nrf2 and resulted in translocation of Nrf2 into the nucleus (Figure 3). The administration of CDDO-EA and CDDO-TFEA at a presymptomatic age enhanced motor performance and extended the survival of SOD1^G93A^ mice, while at a symptomatic age, it only slowed disease progression [178] (Table 2), suggesting that the activation of the Nrf2/ARE signaling pathway may be a useful strategy in the treatment of ALS especially when administered early in the course of the disease.

Another relevant compound is S(+9)-apomorphine, a nonselective dopamine agonist and an activator of the Nrf2/ARE pathway, which has shown the capacity to reduce pathological OS and to improve survival following an oxidative insult in fibroblasts from ALS patients [179]. S(+9)-apomorphine also attenuated motor dysfunction and slowed disease progression in SOD1^G93A^ mice, when administered at 5 mg/kg/day (Table 2) [179]. Another candidate is the green tea polyphenol epigallocatechin-3-gallate (EGCG), a known Nrf2 inducer [180] (Figure 3), that crosses the BBB [181] and that partially protected a motor neuronal cell line expressing SOD1^G93A^ from H_2_O_2_-induced cell death [182]. Oral administration of EGCG (2.9-10 mg/Kg/day) from a presymptomatic stage significantly delayed the onset of disease and extended life span in SOD1^G93A^ mice (Table 2) [183, 184].

### 3.5. Dexpramipexole

Dexpramipexole (RPPX) is the R(+) enantiomer of pramipexole, used in Parkinson's disease, also tested in ALS patients [185, 186]. Dexpramipexole is a lipophilic cation that concentrates into mitochondria, scavenging reactive oxygen and nitrogen species (Figure 3). It was shown to prevent cell death in glutathione-depleted neuroblastoma cells [187, 188] and to block caspase activation in SH-SY5Y neuroblastoma cells treated with methylpyridinium ion (MPP^+^), which induces Parkinson's disease-like neurodegeneration [189]. Treatment with RPPX (100 mg/Kg) in SOD1^G93A^ transgenic mice was shown to prolong survival and preserve motor function [187]. Two-phase I clinical studies in 54 healthy volunteers found that RPPX was safe and well-tolerated in doses up to 150 mg twice a day for 4.5 days [190]. Dexpramipexole (300 mg/day or 50 mg/day for 24 weeks) showed beneficial effects on functional decline and survival in a phase II study in 102 subjects with ALS [191] making it an interesting candidate to include in a multidrug approach for the treatment of ALS. However, in the phase III trial (NCT01281189) with RPPX (150 mg twice daily) in 943 people with ALS, this compound failed to show any efficacy on functional and survival assessment, when compared with placebo control (Table 2) [192]. Considering the discrepant outcomes, Vieira et al. [193] reassessed the effect of RPPX (200 mg/kg) in SOD1^G93A^ transgenic mice but did not recognize any beneficial effects (Table 2). The authors in the latter study argued the lack of balance for sex, age, and weight could justify the previous discrepant results with the same ALS mice model [193].

### 3.6. Melatonin

Melatonin (N-acetyl-5-methoxytryptamine) is a neurohormone secreted by the pineal gland, which has ROS scavenging activity, as well as amphiphilic properties that allow its entrance into both lipophilic and hydrophilic cellular environments [194] (Figure 3). Due to melatonin's antioxidant properties, it has been tested as an experimental drug in different neurodegenerative diseases linked to excessive ROS levels [195]. Besides being a potent free radical scavenger, melatonin also enhances cellular antioxidant potential by stimulating the expression of antioxidant enzymes including SOD, GPx, and GR and by augmenting GSH levels [196]. It was also described that melatonin preserves mitochondrial homeostasis, attenuating free radical generation and promoting mitochondrial ATP synthesis by stimulating the activity of complexes I and IV [197].

In SOD1^G93A^-transgenic mice, the oral administration of melatonin (57–88 mg/kg/day) at a presymptomatic stage delayed disease progression and extended survival [54] (Table 2). The same authors also showed that the rectal administration of 300 mg/day melatonin to 31 sALS patients was well tolerated during an observation period of up to 2 years, reducing circulating serum protein carbonyls, however, without showing any evidences of upregulation of genes encoding antioxidant enzymes [54]. The attenuation of oxidative damage in ALS upon melatonin treatment proved to be safe in humans and suggested the need for further clinical trials to clarify the neuroprotective effect of melatonin in ALS.

More recently, Zhang et al. [198] showed that the administration of melatonin (30 mg/kg) to presymptomatic SOD1^G93A^-transgenic mice significantly delayed disease onset, neurological deterioration, and mortality, which were associated to the inhibition of the caspase-1/cytochrome *c*/caspase-3 pathways and to the reduction of melatonin receptor 1A protein expression. In contrast, Dardiotis et al. [199] showed that the intraperitoneal administration of melatonin (0.5, 2.5, and 50 mg/kg) to presymptomatic SOD1^G93A^-transgenic mice reduced their survival. These authors also reported that, compared to untreated animals, mice treated with melatonin presented an increase in motoneuron loss and in the levels of 4-HNE, a marker of lipid peroxidation, as well as an upregulation of SOD1 expression, suggesting that melatonin exacerbates the disease phenotype in the SOD1^G93A^ mouse ALS model (Table 2), by upregulating toxic SOD1, that overrides its antioxidant and antiapoptotic effects [199]. The fact that the upregulation of mutSOD1 in the SOD1^G93A^ ALS mouse model can influence the beneficial effect of melatonin raises the possibility that this animal model may not be ideal for assessing the neuroprotective properties of melatonin or other molecules with complex antioxidative properties because ALS progression does not always involve SOD1 mutation. Further studies need to be done to understand the mechanisms of action of melatonin and if its antioxidant and antiapoptotic effects can be translated into beneficial effects at the clinical level.

### 3.7. NOX Inhibition

NOX is one of the most important enzymes that regulate ROS production in the CNS, and increasing evidence is showing that NOX inhibition improves neurological disease conditions [200, 201]. In the particular case of ALS, the inactivation of NOX in SOD1^G93A^ transgenic mice has shown to slow disease progression and improve survival [112, 113]. Pharmacological inhibition of NOX using apocynin, a natural organic compound also known as acetovanillone [202] (Figure 3), decreased O_2_^•-^ levels and increased cell viability in MO59J human glioblastoma cells expressing mutSOD1 [114] and decreased ROS levels in primary astrocytes expressing mutSOD1, also restoring motor neuron survival in cocultured hESC-derived motor neurons with human primary astrocytes expressing SOD^G37R^ [203] (Table 2). However, apocynin-mediated NOX inhibition is indirect, involving the presence of myeloperoxidase (MPO) together with H_2_O_2_. These two elements promote the dimerization of apocynin that consequently oxidizes thiols in NOX, being the formation of apocynin dimers necessary to inhibit NOX activity, and not occurring in cells devoid of MPO [204].

Similar to other neurodegenerative diseases, apocynin has been tested in the ALS animal models. In the SOD1^G93A^ transgenic mice, apocynin (30, 150, and 300 mg/kg/day) blocked ROS production, increased the number of neurons in the spinal cord, and prolonged life span compared to wild-type mice [114]. However, Trumbull et al. [205] showed that the administration of apocynin (300 mg/kg/day) had a limited benefit to SOD1^G93A^ mice (Table 2). Although the reasons for this discrepancy have not been clarified, these authors suggested that it could be due to the interference of antibiotics, gender, or the drift in the genetic background resultant from breeding for multiple generations [205]. However, the fact that the treatments with apocynin in mice frequently led to fatal eye infections [113, 114] points to some safety issues regarding this NOX inhibitor. Treatments with apocynin in humans have not been extensively studied; however, some studies were performed in asthmatics receiving nebulized apocynin [206]. Further studies are needed to clarify the functional specificity of apocynin on NOX isomers and to determine a functional dose for therapeutic use. Taking into consideration that mitochondrial ROS and NOX-derived ROS are interrelated, and that an increase in one might lead to the increase in the other [207], the role of NOX-derived ROS production in neurodegenerative diseases needs to be further explored, as a possible strategy of treatment in ALS.

### 3.8. AEOL 10150

AEOL 10150 (manganese [III] tetrakis[N-N′-diethylimidazolium-2-yl]porphyrin) is a manganoporphyrin antioxidant developed by US Aeolus Pharmaceuticals that possesses SOD- and CAT-like activity [208] (Figure 3), being capable of neutralizing O_2_^.-^, H_2_O_2_, and peroxynitrite, and inhibiting lipid peroxidation [209]. The administration of AEOL-10150 at the onset of symptoms markedly prolonged survival in SOD1 transgenic mice [210, 211]. AEOL-10150 decreased 3-nitrotyrosine (3-NT) and malondialdehyde levels in the spinal cord, extended animal survival, provided better preservation of motor neuron architecture, and decreased the level of astrogliosis when administered to ALS mice at symptom onset (at an initial dose of 5.0 mg/kg and a maintenance dose of 2.5 mg/kg/day) [211]. In addition, the use of AEOL-10150 (2.5 mg/kg/day), alone or combined with histone deacetylase inhibitor phenylbutyric acid, was found to significantly enhance motor function and prolong survival [210] (Table 2). Aeolus pharmaceuticals announced that AEOL-10150 was safe and well tolerated in 40 ALS patients and 9 healthy subjects (https://www.accesswire.com/475614/AEOLUS-AEOL-10150-is-Safe-and-Well-Tolerated-in-Phase-1-Study-in-Healthy-Subjects, accessed on 23 July 2020). The same pharmaceutical company also reported that multiple doses of AEOL 10150 up to 2 mg/kg/day over a period of 6.5 days were well tolerated by 12 ALS patients with no serious or clinically significant adverse events (https://www.businesswire.com/news/home/20070322005176/en/Aeolus-Pharmaceuticals-Announces-Successful-Completion-Multiple-Dose, accessed on 23 July 2020).

### 3.9. Edaravone

Edaravone, the active ingredient of Radicut®, is a free radical scavenger widely used in the treatment of cerebral ischemia in Japan [212–214]. Edaravone eliminates lipid peroxides and hydroxyl radicals during cerebral ischemia and exerts a protective effect on the neurons of patients [215, 216]. Although the detailed mechanism of edaravone action is not known, it was proposed that besides its radical scavenger effect, edaravone also inhibits the opening of mitochondrial permeability transition pore (mPTP) in the brain (Figure 3), which may contribute to its neuroprotective effect [217]. Other studies also showed that edaravone reversed the cytotoxic effects of H_2_O_2_ in SH-SY5Y neuroblastoma cells, increasing the expression of Prx2 [218], as an additional neuronal protection mechanism in response to OS. Edaravone also was also shown to promote the antioxidant defense mechanisms by increasing Nrf2, GPx, SOD, HO-1, and NQO1 protein contents (Figure 3), attenuating the effects of traumatic brain injury [219]. In addition, part of the beneficial effects of edaravone can be attributed to its anti-inflammatory capacity [219], which adds to its protective effects in neurons, microglia [220], astrocytes [221], and oligodendrocytes [222].

Preclinical studies demonstrated that edaravone (ranging from 1.5 to 15 mg/kg) improves motor function, slows symptom progression, and attenuates motor neuron degeneration in transgenic SOD1 rodent models of ALS (Table 2) [223, 224].

In an open-label phase II study of 20 patients with ALS, the intravenous administration of edaravone (30 mg or 60 mg/day) was shown to be safe and well-tolerated, slowing disease progression as measured by the revised ALS functional rating scale (ALSFRS-R) score during the six-month treatment period, compared with the six months before the administration of edaravone [225]. Additionally, the same clinical trial also showed that all patients presented a marked reduction in 3-NT in CSF to almost undetectable values, at the end of the six-month treatment period (Table 2) [225], suggesting that the progression delay may be related to the attenuation of OS in ALS patients. A confirmatory double-blind, placebo-controlled study of edaravone in 206 ALS patients (102 edaravone group and 104 placebo group) demonstrated a nonsignificant reduction of ALSFRS-*R* score in patients receiving edaravone over a 24-week treatment period, and the efficacy of edaravone for the treatment of ALS was not demonstrated (NCT00330681) [213]. However, when analyzing only a subgroup of ALS patients (137 patients: 68 edaravone group, 66 placebo group) with scores of 2 or more on all items of ALSFRS-R, forced vital capacity of 80% at baseline, and disease duration of 2 years or less, significant differences were observed in the ALSFRS-R score after treatment with edaravone (60 mg intravenous) compared with placebo, suggesting a potential benefit of edaravone in a well-defined subset of ALS patients (NCT01492686, Table 2) [5]. Additionally, in the open-label 24-week extension period, edaravone maintained its beneficial effects throughout 48 weeks in ALS patients, with no new or cumulative safety concerns (NCT01492686) [226].

Currently, edaravone is approved for use as a treatment for ALS in Japan and South Korea, having been also approved by the FDA in May 2017 [227], although its mechanism of action remains unclear. A phase I trial of an oral formulation of edaravone (TW001) developed by the Treeway company has returned positive results, proving to be safe and well-tolerated with the oral formulation (http://www.cphi-online.com/treeway-announces-positive-data-from-two-separate-news038315.html, accessed on 23 July 2020). Two recent clinical trials, sponsored by Mitsubishi Tanabe Pharma Development America, Inc., are in progress to evaluate the pharmacokinetics of single doses of edaravone oral suspension in ALS patients with gastrostomy (NCT04254913, phase I), as well as to evaluate the long-term safety and tolerability of oral edaravone in subjects with ALS over 24 and 48 weeks (NCT04165824, phase III).

### 3.10. Riluzole

Riluzole is a benzothiazole with antiglutaminergic properties which has shown a modest survival benefit (about 3 months) in patients at a dosage of 100 mg/day without showing any effect in muscle strength [228, 229]. Although the precise neuroprotective mechanisms of riluzole are not completely understood, it has been proposed that it has multiple effects beyond the inhibition of glutamate release in presynaptic terminals through the blockage of voltage-gated sodium channels [230]. It has been demonstrated that riluzole also affects the chloride, calcium, and potassium channels and interferes with intracellular events that follow transmitter binding at excitatory amino acid receptors [230, 231], which can include OS [232]. However, other studies have also evidenced some antioxidant properties of this compound, mediated by the inhibition of protein kinase C [233] and phospholipase A activities [234] that consequently attenuate a broad spectrum of oxidative damage. Consistent with its antioxidative effects, it is also been shown that riluzole also decreases methylmercury-induced OS by promoting the elevation of GSH synthesis through the activation of glutamate transporters and the increase of intracellular glutamate levels, which is a GSH precursor [123, 235]. Based on these different studies, it is possible that the beneficial effect of riluzole may be due to a combined action on different targets that still remain largely unclear. Thus, although riluzole treatment prevents cell death and controls increased ROS levels in parental SH-SY5Y cells, it was shown to be ineffective in reversing ROS effects in SH-SY5Y cells carrying the G93A SOD1 mutation [236],suggesting that riluzole is unable to reverse chronic oxidative damage. This situation is in agreement with the fact that this compound does not present significant benefit on lifespan and motor performance in SOD1^G93A^ transgenic mice [237]. The fact the riluzole has a direct antioxidant effect against acute OS, but not against RNS [236], also supports the hypothesis that combined treatment with edaravone may be more effective in treating ALS, since edaravone has the capacity to reduce RNS [225, 238].

### 3.11. NAD^+^/SIRT1 Modulators

NAD^+^ plays a key role in many redox reactions in the cells, being involved in many processes including signaling pathways, gene expression, DNA repair, and mitochondrial metabolism [239]. NAD^+^ is also a cosubstrate for sirtuins (SIRTs), a family of signaling proteins involved in the regulation of cellular metabolic status, playing a key role in several processes such as mitochondrial function, DNA repair, and also activating metabolic pathways responsible for the detoxification of ROS (e.g., SOD, CAT, and isocitrate dehydrogenase 1) [240]. Sirtuins regulate peroxisome proliferator-activated receptor gamma coactivator 1-alpha (PGC1*α*), which affects mitochondrial biogenesis, activity, and dynamics [241, 242] and is considered a promising therapeutic target for ALS [243]. Decreased SIRT1 levels have been found in postmortem tissues from ALS patients [244] and intraperitoneal injection of the SIRT1 activator resveratrol resulted in a significant improvement in both symptoms and survival of SOD1^G93A^ mice [245]. In addition, SIRT3 was reported to protect against mitochondrial fragmentation and neuronal cell death induced by SOD1^G93A^ overexpression in cultured rat spinal cord motor neurons [246].

Therapeutic strategies based on NAD^+^ precursors, including nicotinamide (NAM), nicotinic acid (NA), nicotinamide riboside (NR), and nicotinamide mononucleotide (NMN), [247] have been proposed in ALS [248] (Figure 3). NMN and NR (5 mM for 24 h) were shown to increase total and mitochondrial NAD^+^ content in astrocytes from SOD^G93A^ mice, which was associated with an increase in OS resistance and reversal of astrocyte toxicity towards co-cultured motor neurons [248] (Table 2). The effects of modulation of NAD^+^ availability in SOD1^G93A^ mice were also tested, using two strategies: supplementation with NR and ablation of a NAD^+^-consuming enzyme (CD38) [249]. NR was found to delay motor neuron degeneration, whereas CD38 ablation was not protective [249]. The same study also found that the expression of NMNAT2 (nicotinamide mononucleotide adenylyl transferase 2, involved in NAD^+^ synthesis) and SIRT6 was decreased in the spinal cord of ALS patients, suggesting a deficit of this neuroprotective pathway in humans and highlighting the therapeutic potential of increasing NAD^+^ levels in ALS [249]. Since NAD^+^ supplementation is known to promote neural stem cells/neuronal precursor cells (NSCs/NPCs) pool maintenance, another study wanted to determine if the administration of NR could enhance the proliferation and migration of NSCs/NPCs in ALS [250]. SOD1^G93A^ transgenic and wild-type mice were treated with 400 mg/kg/day, starting at 50 days of age, which was found to improve the adult neurogenesis in the brain of SOD1^G93A^ mice [251]. This was associated with the activation of mitochondrial unfolded protein response (UPRmt) signaling and modulation of mitochondrial proteostasis, which can ameliorate misfolded protein accumulation. Increasing total NAD^+^ content in astrocytes using NMN (5 mM and 24 h) was reported to induce the activation of Nrf2 and upregulation of the antioxidant proteins HO-1 and sulfiredoxin 1 (SRXN1), mediated by SIRT6 [252].

A clinical trial based on modulation of NAD^+^/sirtuins in ALS used EH301, which is a combination of two active compounds (1-(beta-D-ribofuranosyl)nicotinamide chloride and 3,5-dimethoxy-4′-hydroxy-trans-stilbene) from Elysium Health, that were proposed to act synergistically to increase NAD^+^ levels and support SIRT activity [253]. This was a single-center, prospective, double-blind, randomized, placebo-controlled pilot study (NCT03489200), in which the efficacy of EH301 (1200 mg) was tested in ALS patients. The results of this trial showed that EH301 significantly slowed the progression of ALS compared to placebo, also showing improvements in several key outcome measures compared with baseline [253] (Table 2). A phase II clinical trial has been planned to expand the scope of the original trial with EH301, using over-the-counter antioxidants such as CoQ10, vitamin E, NAC, and L-cystine at safe dosages (NCT04244630). This study is expected to be completed in December 2021.

### 3.12. Mitochondria-Targeted Antioxidants

The mitochondria-targeted antioxidant 10-(60-ubiquinonyl) decyltriphenylphosphonium (MitoQ) comprises a triphenylphosphonium (TPP) functional group conjugated to an ubiquinone antioxidant moiety [254]. MitoQ crosses biological membranes, accumulates inside mitochondria driven by the transmembrane electric potential [255], and effectively prevents mitochondrial oxidative damage [254, 256] (Figure 3). Within mitochondria, the ubiquinone moiety of MitoQ is reduced to its active ubiquinol form, protecting mitochondria against oxidative damage that could be derived from the leakage of electrons [256]. Reactions with a variety of oxidants readily oxidize ubiquinol to ubiquinone, which is quickly reduced back to ubiquinol by the respiratory chain [257] and is thus continually recycled. A protective effect of MitoQ was described in chronic hepatitis C patients, by decreasing liver damage [258], as well as in some neurodegenerative diseases, including Parkinson's [259, 260] and Alzheimer's [261, 262] diseases, by decreasing the oxidative damage. However, very disappointing results were obtained when MitoQ was used in a phase II clinical trial for the treatment of Parkinson's disease (NCT00329056-Antipodean Pharmaceuticals, Inc.) [263]. Despite these negative results, MitoQ treatment reduced nitroxidative stress and mitochondrial dysfunction in SOD1^G93A^-expressing astrocytes, reducing the toxicity to motor neurons in cocultures [264]. Also, the administration of MitoQ (500 *μ*M) improved the ALS phenotype in the SOD1^G93A^ mice, slowing the decline of mitochondrial function in both the spinal cord and quadriceps muscle and increasing the life span of affected animals [265]. Importantly, the same authors also described a marked reduction of nitroxidative markers and pathological signs in the spinal cord of MitoQ-treated animals, as well as the recovering of the neuromuscular junctions associated with a significant increase in hindlimb strength [265] (Table 2). These results, associated to the fact that MitoQ rapidly crosses the BBB [261] and is well-tolerated in both animals and humans [258], with nauseas as the most common side effect [266], pointed out mitochondria-directed antioxidants as a possible strategy to delay ALS symptoms, that deserves to be further developed.

Another mitochondria-targeted antioxidant tested in ALS models was the mitochondria-targeted carboxy-proxyl (Mito-CP), which also comprises TPP cation covalently coupled to carboxy-proxyl nitroxide and, similarly to MitoQ, accumulates into the mitochondria [267]. Low doses of Mito-CP (1-10 nM) effectively prevented the death of motor neurons expressing SOD1^G93A^ induced by nerve growth factor (NGF), which involved an increase in mitochondrial O_2_^•-^ [268] (Figure 3), and also avoided mitochondrial dysfunction in SOD1^G93A^ astrocytes, decreasing O_2_^•-^ levels, and restoring motor neuron survival [264] (Table 2). However, additional studies should be performed with other types of mitochondriotropic compounds, with lower toxicity and higher therapeutic efficacy.

The cell-permeable antioxidant peptide SS31 (D-Arg-Dmt-Lys-Phe-NH_2_), which targets the IMM and protects against mitochondrial oxidative damage (Figure 3), was also tested by Petri et al. [269] in *in vitro* and *in vivo* models of ALS associated with SOD1^G93A^ mutations. These authors showed that SS-31 (1 *μ*M) protected cells against cell death induced by H_2_O_2_ in N2a mouse neuroblastoma cells transfected either with wild type or mutSOD1. The administration of SS-31 (5 mg/kg/day) to SOD1^G93A^ mice at the presymptomatic stage led to decreases in cell loss, lipid peroxidation and protein nitration (4-HNE and 3-NT) markers in the lumbar spinal cord. Moreover, it significantly improved the survival and motor performance compared to controls [269] (Table 2). The capacity of SS-31 to inhibit the mPTP and cytochrome *c* release induced by the addition of calcium in isolated liver mitochondria [270], and its ability to protect against the loss of mitochondrial potential, and apoptosis induced by tert-butyl hydroperoxide in N2A and SH-SY5Y cells [271], suggests that this antioxidant can be a very interesting therapeutic strategy to treat neuronal damage in ALS, and this needs to be explored in the future.

## 4. Conclusions

Several studies have been adding strong evidence to the role of OS mechanisms in ALS that culminate in mitochondrial dysfunction and cell damage and contribute to neurodegeneration. If it is accepted that the excessive ROS production is a common pathological feature in ALS patients, there are doubts whether oxidative damage represents a primary cause or a secondary consequence of this disease and what is the real contribution of OS in ALS progression, considering the different subtypes of patients. Although the mechanisms of OS and mitochondrial dysfunction represent promising therapeutic targets to slow the disease progression, it is of utmost importance to characterize the different OS profiles present in different types of patients (e.g., identify different OS mechanisms associated with different mutations), in order to develop personalized therapies that allow retarding the progression of the disease according to the OS profiles of patients.

Although several antioxidants have shown beneficial effects in ALS animal models, they have failed to show any meaningful therapeutic benefit in ALS patients. There are different reasons for the lack of beneficial effects of antioxidant therapies in ALS patients that should be considered in the future. Some reasons evidenced are the lack of proper blinding measurements, uniform exclusion criteria, or statistical power associated with the use of a small number of samples per group in animal assays that can lead to false-positive results and confounding biological results [272]. These issues should be avoided by following programmed experimental designs based on the guidelines for preclinical animal research in ALS [273]. Moreover, the low CNS bioavailability of some of the antioxidants may limit their pharmacological effects, being necessary to invest more in compounds that can cross the BBB. Another reason may be the fact that most antioxidants have been exclusively tested in mutSOD1 animal models, being the mutSOD1 representative of a small percentage of patients [274, 275]. To overcome this issue, it is necessary to invest more on the development of ALS models that can be representative of the different subtypes of this disease, which can support preclinical trials before proceeding to clinical trials. Preclinical trials using other animal models of the disease should be done in parallel with the common mutSOD1 models, including C9orf72 and TDP-43 mice models that represent the most prevalent mutation in ALS and the formation of ubiquitinated TDP-43 cytoplasmic inclusions that are expressed in the majority of patients, respectively [276, 277]. Although less characterized than the mutSOD1 models and with some construct validity limitations, both C9orf72 and TDP-43 mice models develop many features of ALS [276, 277] that can be of extreme usefulness in the future to complement the experiments with the traditional mutSOD1 models. Complementarily, the use of preclinical assays that are not based on specific mutations models should be considered, as for example the use of iPSCs from sALS and fALS patients (with or without specific mutations) that can be differentiated in MNs, astrocytes, and microglial cells [278] and represent a valuable tool for screening different compounds [279]. Another reason that can also explain this failure is the fact that these compounds have been generally tested in animal models in presymptomatic stages of the disease that do not represent the stage in which patients are diagnosed [274]. It is necessary to establish limits of the disease progression, based on OS profiles, that allow understanding until what stage of the disease a certain compound may have any beneficial effect.

Among the different antioxidant strategies described in Section 3, there are two that should be explored in the future due to their capacity to control OS mechanisms and improve the mitochondrial function. The first example is the case of NAD^+^/SIRT1 modulators that have shown capacity to increase mitochondrial OS resistance and protect against mitochondrial dysfunction in ALS models, as well as to significantly slow the disease progression in ALS patients, tested in a phase I clinical trial (NCT03489200) [253]. To confirm these results, a clinical trial phase II is already planned, which is expected to be complete by the end of 2021 (NCT04244630). The second strategy of great importance is the development of mitochondria-targeted antioxidants that have shown a capacity to accumulate inside mitochondria, prevent mitochondrial oxidative damage, and attenuate mitochondrial dysfunction. Although the administration of MitoQ has shown very promising results in the SOD1^G93A^ mice by slowing the decline of mitochondrial function in both the spinal cord and quadriceps muscle, by recovering the neuromuscular junctions associated with a significant increase in hindlimb strength, and by increasing the life span of the affected animals [265], its disappointing results in a phase II clinical trial for the treatment of Parkinson's disease (NCT00329056-Antipodean Pharmaceuticals, Inc.) [263] evidenced also the necessity to develop other types of mitochondriotropic compounds, with lower toxicity and higher therapeutic efficacy that may afterwards be tested in ALS models.

Altogether, the present review shows the need to invest on the characterization of OS profiles which are representative of each subtype of patient, permitting the development of personalized therapies based on the differential OS mechanisms that characterize different subtypes of patients. This approach will allow understanding what are the characteristics of certain antioxidants that can have beneficial effects on different subtypes of patients and help to understand what is the disease progression window at which a compound may have beneficial effects.

## Figures and Tables

**Table 1:** Representative studies that demonstrate the association of specific genetic alterations with oxidative stress in ALS.

Altered gene	Genetic alterations	Experimental model	Observed effects on oxidative stress makers	Reference
SOD1	Mutation: G93A	(i) Transgenic mice	(i) Reduced GSH in the spinal cord and motor neuron cells that correlates with apoptosis-inducing factor translocation, caspase 3 activation, and motor neuron degeneration during ALS-like disease onset and progression	[70]

SOD1	Mutations: A4V, G37R, H48Q, H80R, G85R, D90A, G93A, D124V, D125H, E138*Δ*, S134N, H46R	(i) NSC-34 motor neuron-like cell line	(i) MutSOD1s lowered the GSH/GSSG ratio in mitochondria of cells	[69]

SOD1	Mutations: G1H, G1L, A4V, H46R, G93A, frame-shift 126 mutation	(i) Motor neurons from 40 sALS and 5 mutated SOD1 sALS patients (frame-shift 126 mutation and A4V) (ii) Transgenic rats (H46R/G93A) (iii) Transgenic mice (G1H/G1L-G93A)	(i) The number of motor neurons with negative expression of antioxidant enzymes (Prxll and GPxl) increased with ALS disease (ii) Neurons with higher expression of Prxll and GPxl were less susceptible to oxidative stress	[98]

TDP-43	Mutations: M33V, Q331K	(i) TDP-43Q331K mice (ii) Primary astrocyte cultures from TDP-43Q331K mice (iii) Fibroblasts from pre- and postsymptomatic ALS patient fibroblasts harboring a TDP-43M337V mutation	(i) Increased transcript expression of Nrf2 signaling-related genes (NFE2L2, HMOX1, GCLM, and NQO1) in the spinal cord of transgenic mice (ii) No change in protein expression levels of HO-1, GCLM, GPx1, and NQO1 antioxidant proteins in transgenic mice (impaired protein translation of antioxidants) (iii) Decreased total GSH levels in fibroblasts from pre- and postsymptomatic patients (iv) Decreased total GSH levels in primary astrocytes from transgenic mice	[116]

TDP-43	Mutation: M337V	(i) NSC-34 motor neuron-like cell line	(i) Decreased nuclear translocation of Nrf2, total Nrf2, cytoplasmic Nrf2, and downstream phase II detoxifying enzyme (NQO1) (ii) Increased lipid peroxidation products	[115]

TDP-43	Mutations: Q331K, M337V	(i) NSC-34 motor neuron-like cell line	(i) Mitochondrial dysfunction, oxidative damage, and nuclear accumulation of Nrf2 in cells (ii) Downregulation of HO-1, that could not be restored by sulforaphane (iii) Reduction of LDH and lipid peroxidation products by sulforaphane	[117]

C9orf72	GGGGCC hexanucleotide repeat expansion in noncoding region	(i) iPSC-derived astrocytes from C9orf72-mutated fALS patients and nonaffected donors	(i) Decreased secretion of antioxidant proteins (SOD1, SOD2, and GSH) in mutant C9orf72 astrocytes (ii) Increased ROS levels in mutant C9orf72 astrocytes (iii) Conditioned media of mutant C9orf72 astrocytes increased ROS levels in wild type motor neurons (iv) Oxidative stress was increased in an age-dependent manner (v) poly(GR) in C9orf72 neurons compromises mitochondrial function and causes DNA damage in part by increasing oxidative stress	[129]

C9orf72	GGGGCC hexanucleotide repeat expansion in noncoding region	(i) iPSCs-derived motor neurons isolated from C9orf72-mutated fALS patients (ii) iPSC-derived control neurons expressing (GR)80 and dipeptide repeat (DPR) protein	(i) Increased mitochondrial ROS levels cause DNA damage in both models (ii) Prevention of DNA damage by an antioxidant (Trolox)	[128]

ANG	Human wild type ANG (wANG) and its variant K40I (mANG)	(i) SH-SY5Y neuroblastoma cells and NSC-34 motor neuron-like cell line	(i) wANG prevented cell death under H_2_O_2_-induced oxidative stress (ii) Increased hydrogen peroxide-induced cell damage in mutant ANG motor NSC-34 neuron-like cell line	[131]

fALS: familial ALS; GCLM: glutamate-cysteine ligase modifier subunit; GPX1: glutathione peroxidase-l: HMOX1: heme oxygenase-1; iPS: induced pluripotent stem cell; LDH: lactate dehydrogenase; NQO1: NAD(P)H quinone dehydrogenase 1; PrxII: peroxiredoxin-ll; sALS: sporadic ALS.

**Table 2:** Preclinical and clinical studies with different antioxidant therapies for ALS.

Antioxidant	Preclinical animal or cellular model/ clinical trial	Dose/concentration (treatment time)	Effects	Reference
Vitamin E	SOD1^G93A^ transgenic mice	200 UI/Kg (starting at 30 days of age)	(i) Slowed disease progression, delayed (ii) Disease onset, did not affect survival time	[140]
	RCT	500 mg twice daily—5000 mg/day (18 months)	(i) Did not affect the quality of life (ii) Did not affect survival time (iii) Slowed ALS progression	[147–149]
N-Acetyl-L-cysteine (NAC)	SH-SY5Y cells with SOD1^G93A^	1 mM (24 h)	(i) Reduction mROS (ii) Increased ATP levels (iii) Increased viability	[155]
	SOD1^G93A^ transgenic mice	2 mg/kg/day (from 4-5 weeks of age)	(i) Prolonged the survival time (ii) Improved motor performance	[153]
	RCT	50 mg/kg s.c. infusion (12 months)	(i) Did not affect survival time (ii) Did not affect disease progression	[156]
Coenzyme Q10	SOD1^G93A^ transgenic mice	200 mg/kg daily (from 50 days after birth)	(i) Prolonged the survival time	[159]
	SOD1^G93A^ transgenic mice	800 mg/kg/daily (from symptom onset)	(i) No effect on survival time	[160]
	RCT, NCT00243932	2700 mg/kg, three times daily (9 months)	(i) No significant differences between treatment and placebo groups	[164, 165]
Nrf2/ARE modulators				
WN1316	SOD1^H46R^ and SOD1^G93A^ transgenic mice	1-100 *μ*g/kg/day (from 21–22 weeks of age)	(i) Improved motor function, prolonged survival time; reduced motor neuron loss, gliosis, and oxidative damage	[170]
Dimethoxy curcumin	NSC-34 cell lines transfected with M337V or Q331K mutant TDP-43	15 *μ*M (3 days)	(i) Improved mitochondrial dysfunction	[173]
Nanocurcumin (SinaCurcumin)	RCL	80 mg/day (3 months)	(i) Improved the probability of survival time	[174]
Curcumin (Brainoil)	RCL	600 mg/day (6 months)	(i) Slowed disease progression, reduced oxidative stress	[175]
CDDO-EA, CDDO-TFEA	SOD1^G93A^ transgenic mice	80 mg/kg/day (starting at 30 days of age or from the onset of the disease)	(i) At presymptomatic age: enhanced motor performance and prolonged survival time (ii) At symptomatic age: slowed disease progression	[178]
S(+9)-apomorphine	Fibroblasts from ALS patients		(i) Reduced oxidative stress, improved survival after oxidative insult	[179]
	SOD1^G93A^ transgenic mice	5 mg/kg/day s.c. (from day 21 until end)	(i) Enhanced motor performance, slowed disease progression	[179]
EGCG	Neuronal-differentiated VSC 4.1 cells with SOD1^G93A^	20, 40, 50, 100 *μ*M (2 h)	(i) Reduced H_2_O_2_-induced cell death	[182]
	SOD1^G93A^ transgenic mice	2.9 *μ*M/g/day 10 mg/kg/day (from presymptomatic stage)	(i) Delayed disease onset and prolonged survival time	[183, 184]
RPPX	SOD1^G93A^ transgenic mice	100 mg/kg/day, p.o (from day 45)	(i) Enhanced motor performance and prolonged survival time	[187]
	SOD1^G93A^ transgenic mice	200 mg/kg/day (from day 55 until 180 days)	(i) No effect was observed on disease progression or survival	[193]
	RCT, phase II	50 mg/day or 300 mg/day (24 weeks)	(i) Beneficial effects on functional decline and survival	[191]
	RCT, phase III (NCT01281189)	150 mg/twice daily (12-18 months)	(i) Did not show any efficacy on functional and survival assessment	[192]
Melatonin	SOD1^G93A^ transgenic mice	57–88 mg/kg/day, p.o. (from presymptomatic stage)	(i) Slowed disease progression and prolonged survival time	[54]
	SOD1^G93A^ transgenic mice	30 mg/kg/day, i.p. (from six weeks of age)	(i) Delayed disease onset, slowed disease progression, and neurological deterioration and mortality	[198]
	SOD1^G93A^ transgenic mice	0.5, 2.5 and 50 mg/kg (from presymptomatic stage)	(i) Increased the motoneuron loss and lipid peroxidation, reduced survival time	[199]
NOX	SOD1^G93A^ transgenic mice	Deletion of NOX	(i) Slowed disease progression and prolonged survival time	[112, 113]
Apocynin	MO59J glial cells and SH-SY neuronal cells overexpressing mutant SOD1	100 *μ*M	(i) Decreased O_2_^.-^ levels and increased cell viability	[114]
	SOD1^G93A^ transgenic mice	30, 150, and 300 mg/kg/day (from 2 weeks of age)	(i) Decreased ROS levels, increased neurons in the spinal cord, prolonged survival time, and slowed disease progression	[114]
	SOD1^G93A^ transgenic mice	300 mg/kg/day (from 21 days of age)	(i) Failed to significantly prolong survival time	[205]
	Cocultured hESC-derived motor neurons with human primary astrocytes expressing SOD1^G37R^	300 *μ*M (48 h pretreatment)	(i) Prevented motor neuron loss (ii) Decreased ROS levels	[203]
AEOL10150	SOD1^G93A^ transgenic mice	Initial dose of 5.0 mg/kg and a maintenance dose of 2.5 mg/kg/day i.p. (from the onset of the disease)	(i) Reduced oxidative stress, enhanced motor performance, prevented motor neuron loss, prolonged survival time	[210]
	SOD1^G93A^ transgenic mice	2.5 mg/kg/day i.p. (from the onset of the disease)	(i) Reduced astrogliosis, prevented motor neuron loss, prolonged survival time	[211]
Edaravone	SH-SY5Y cells	25 *μ*M (8 h)	(i) Reduced H_2_O_2_-induced cell death	[218]
	SOD1^G93A^ transgenic mice	5 mg/kg/day and 15 mg/kg/day i.p. (from the onset of the disease)	(i) Slowed motor decline, prevented motor neuron loss, slowed disease progression	[224]
	SOD1^H46R^ transgenic rats	1.5 or 3.0 mg/kg/h i.v. continuous infusion (1 h per day) for 2 days, followed by a 2-day holiday (y from 18 weeks of age to the day of loss of righting reflex)	(i) Improved motor function	[223]
	Open-label phase II	30 mg or 60 mg/day i.v. (6 months) two weeks of administration followed by a two-week observation period (4 weeks cycle repeated six times)	(i) Slowed disease progression (using ALSFRS-R) (ii) Reduced 3-NT levels in cerebrospinal fluid	[225]
	RCT, phase III, NCT00330681	60 mg/day i.v. during 60 min (24-week treatment)	(i) Did not significantly reduce the ALSFRS-R score. Significant differences observed when analyzing a subgroup of patients (scored of at least 2 points on all 12 items of ALSFRS-R, forced vital capacity of 80% or more, disease duration of 2 years or less)	[213]
	RCT, phase III, NCT01492686	60 mg/day i.v. during 60 min (24-week treatment)	(i) Slowed disease progression (using ALSFRS-R) in a well-defined population of ALS patients	[5]
Riluzole	Mixed mouse cortical culture	30 or 100 *μ*M (30 min treatment)	(i) Blocked phorbol 12-myristate	[233]
	Cortical cultures	1-30 *μ*M (24 h treatment)	(i) Attenuated neuronal death induced by 30 *μ*M kainate or NMDA, but not that by 100 *μ*M NMDA (ii) Attenuated nonexcitotoxic oxidative injury induced by exposure to FeCl_3_ in the presence of MK-801 and CNQX (iii) Reduced Fe^3+^-induced lipid peroxidation, and inhibited cytosolic phospholipase A2	[234]
	Rats	21.35 *μ*mol/kg (every two days, lasting for 4 weeks)	(i) Antagonized methylmercury-induced oxidative through elevation of GSH synthesis by activating of glutamate transporters	[235]
	Human SH-SY5Y neuroblastoma cells	1-10 *μ*M	(i) Counteracted the effects of H_2_O_2_ exposure (ii) Demonstrated direct antioxidant defense capacities against acute oxidative, but not on nitrosative stress	[236]
	SOD1^G93A^ model, the TDP-43^A315T^ model, and FUS (1-359) model	22 mg/kg (in drinking water from symptom onset)	(i) Had no significant benefit on lifespan in any of the ALS mouse models tested	[237]
NAD^+^/SIRT1 modulators				
NMN and NR	SOD1^G93^ mice astrocytes	5 mM (24 h pretreatment)	Increased total and mitochondrial NAD^+^ content in, increased oxidative resistance and reversal of astrocyte toxicity towards cocultured motor neurons	[248]
NR	SOD1^G93A^ transgenic mice	400 mg/kg/day	NR supplementation delayed motor neuron degeneration, decreased markers of neuroinflammation in the spinal cord, modified muscle metabolism, and prolonged survival time	[249]
EH301	RCT, NCT03489200	1200 mg (4 months)	Slowed the progression of ALS (using ALSFRS-R)	[253]
Mitochondria-Targeted Antioxidants				
MitoQ				
	SOD1^G93A^ rat astrocytes	10–100 nM (24 h pretreatment)	Reduced nitroxidative stress and mitochondrial dysfunction. Restored motor neuron survival in cocultures	[264]
	SOD1^G93A^ motor neurons	1-10 pM (48 h pretreatment)	Prevented NGF-induced neuron loss	[268]
	SOD1^G93A^ transgenic mice	500 *μ*M (from 90 days of age)	Slowed decline of mitochondrial function, reduced nitroxidative markers and pathological signs in the spinal cord, neuromuscular junctions were recovered associated with a significant increase in hindlimb strength, prolonged survival time	[265]
Mito-CP	SOD1^G93A^ rat astrocytes	10–100 nM (24 h pretreatment)	Reduced nitroxidative stress and mitochondrial dysfunction. Restored motor neuron survival in cocultures	[264]
	SOD1^G93A^ motor neurons	100-1000 pM (48 h pretreatment)	Prevented NGF-induced neuron loss	[268]
SS-31	N2a cells overexpressing SOD1^G93A^	1, 10, or 100 *μ*M (6 h pretreatment)	Reduced H_2_O_2_-induced cell death	[269]
	SOD1^G93A^ transgenic mice	5 mg/kg/day i.p. (from 30 days of age)	Decreased cell loss, decreased markers of oxidative stress in the lumbar spinal cord, improved motor function, and prolonged survival time	[269]

RTC: double-blind randomized controlled trial; mROS: mitochondrial ROS production; s.c: subcutaneous; p.o.: oral; i.p: intraperitoneal; i.v.: intravenous; WN1316:2-[mesityl(methyl)amino]-N-[4-(pyridin-2-yl)-1H-imidazol-2-yl] acetamide trihydrochloride; CDDO-EA: 2-cyano-3,12-dioxooleana-1,9-dien-28-oic acid ethylamide; CDDO-TFEA:2-cyano-3,12-dioxooleana-1,9-dien-28-oic acid trifluoroethylamide; EGCG: epigallocatechin-3-gallate; RPPX: dexpramipexole; NOX: nicotinamide adenine dinucleotide phosphate oxidase; ROS: reactive oxygen species; AEOL10150: manganese [III] tetrakis[N-N′-diethylimidazolium-2-yl]porphyrin; Edaravone: 3-methyl-1-phenyl-2-pyrazolin-5-one; ALSFRS-R: revised ALS functional rating scale; 3-NT: 3-nitrotyrosine; NMDA: N-methyl-D-aspartate; CNQX: 6-cyano-7-nitroquinoxaline-2,3-dione; MK-801: (5R,10S) (+)-5-methyl-10,11-dihydro-5H-dibenzo[a,d]cyclohepten-5, 10-imine hydrogen maleate; MitoQ: [10-(4,5-dimethoxy-2-methyl-3,6-dioxo-1,4-cyclohexadien-1-yl)decyl]triphenylphosphonium methane sulfonate; Mito-CP: mitochondria-targeted carboxy-proxyl; SS-31: cell-permeable peptide antioxidant D-ArgDmt-Lys-Phe-NH2; NMN and NR: nicotinamide mononucleotide and nicotinamide riboside.

## Abbreviations

AEOL 10150: Manganese [III] tetrakis[N-N′-diethylimidazolium-2-yl]porphyrin
ALS: Amyotrophic lateral sclerosis
ALSFRS-R: Revised amyotrophic lateral sclerosis functional rating scale
ANG: Angiogenin
ARE: Antioxidant response element
BBB: Blood-brain barrier
C9orf72: Chromosome 9 open reading frame 72
CAT: Catalase
CD38: Cluster of differentiation 38 (NAD^+^-consuming enzyme)
CDDO-EA: 2-Cyano-3, 12-dioxooleana-1,9-dien-28-oic acid ethylamide
CDDO-TFEA: 2-Cyano-3, 12-dioxooleana-1,9-dien-28-oic acid-trifluoroethylamide
CNS: Central nervous system
CoQ10: Coenzyme Q10
CSF: Cerebrospinal fluid
CYP: Cytochrome P450
DPR: Dipeptide repeat
EGCG: Epigallocatechin-3-gallate
fALS: Familial amyotrophic lateral sclerosis
FUS: Fused in sarcoma
GPx: Glutathione peroxidase
GR: Glutathione reductase
GSH: Glutathione
Keap1: Kelch ECH-associating protein 1
H_2_O_2_: Hydrogen peroxide
4-HNE: 4-Hydroxynonenal
HO•: Hydroxyl radical
HO-1: Heme oxygenase
hnRs: Heterogeneous nuclear ribonucleoproteins
IMM: Inner mitochondrial membrane
IMS: Intermembrane mitochondrial space
iPSCs: Induced pluripotent stem cells
Keap1: Kelch ECH-associating protein 1
MitoQ: 10-(60-Ubiquinonyl) decyltriphenylphosphonium
Mito-CP: Triphenyl-phosphonium-carboxy-proxyl
mPTP: Mitochondrial permeability transition pore
mutSOD1: SOD1 mutation
MM: Mitochondrial matrix
MNs: Motor neurons
MPO: Myeloperoxidase
MPP^+^: 1-methyl-4-phenylpyridinium
NA: Nicotinic acid
NAC: N-Acetyl-L-cysteine
NADPH: Nicotinamide adenine dinucleotide phosphate
NAM: Nicotinamide
NF-kB: Nuclear factor kappa-light-chain-enhancer of activated B cells
NGF: Nerve growth factor
NMN: Nicotinamide mononucleotide
NMNAT2: Nicotinamide mononucleotide adenylyl transferase 2
NOX: Nicotinamide adenine dinucleotide phosphate oxidase
NPCs: Neuronal precursor cells
NQO1: NAD(P)H quinone dehydrogenase 1
NR: Nicotinamide riboside
Nrf2: Nuclear factor erythroid -2-related factor 2
NSCs: Neural stem cells
3-NT: 3-Nitrotyrosine
O_2_^•^: Superoxide radical anion
8-OHdG: 8-hydroxy-2′-deoxyguanosine
OS: Oxidative stress
PGC-1*α*: Peroxisome proliferator-activated receptor gamma coactivator 1-alpha
Poly-GA: Poly-glycine-alanine
Poly-GP: Poly-glycine-proline
Poly-GR: Poly-glycine-arginine
Poly-PA: Poly-proline-alanine
Poly-PR: Poly-proline-arginine
PON: Paraoxonase
Prxl: Peroxiredoxin-l
Rac1: Ras-related C3 botulinum toxin substrate 1
ROS: Reactive oxygen species
RPPX: Dexpramipexole
sALS: Sporadic amyotrophic lateral sclerosis
SIRT: Sirtuin
SOD1: Cu^2+^/Zn^2+^ superoxide dismutase type-1
SRXN1: Sulfiredoxin 1
SS-31: D-ArgDmt-Lys-Phe-NH2
TARDBP: TAR DNA-Binding
TDP-43: TAR DNA-Binding Protein 43
TPP: Triphenylphosphonium
Trx: Thioredoxin
UBQLN2: Ubiquilin 2
UPRmt: Mitochondrial unfolded-protein response
WN1316: 2-[mesityl(methyl)amino]-N-[4-(pyridin-2-yl)-1H-imidazol-2-yl] acetamide trihydrochloride.

## References

[1] Tafuri F., Ronchi D., Magri F., Comi G. P., Corti S. (2015). SOD1 misplacing and mitochondrial dysfunction in amyotrophic lateral sclerosis pathogenesis. *Frontiers in Cellular Neuroscience*.

[2] Zarei S., Carr K., Reiley L. (2015). A comprehensive review of amyotrophic lateral sclerosis. *Surgical Neurology International*.

[3] Smith E. F., Shaw P. J., De Vos K. J. (2019). The role of mitochondria in amyotrophic lateral sclerosis. *Neuroscience Letters*.

[4] Tanaka M., Sakata T., Palumbo J., Akimoto M. (2016). A 24-week, phase III, double-blind, parallel-group study of edaravone (MCI-186) for treatment of amyotrophic lateral sclerosis (ALS) (P3.189). *Neurology*.

[5] The Writing Group (2017). Safety and efficacy of edaravone in well defined patients with amyotrophic lateral sclerosis: a randomised, double-blind, placebo-controlled trial. *The Lancet Neurology*.

[6] McCombe P. A., Henderson R. D. (2010). Effects of gender in amyotrophic lateral sclerosis. *Gender Medicine*.

[7] Valdmanis P. N., Rouleau G. A. (2008). Genetics of familial amyotrophic lateral sclerosis. *Neurology*.

[8] Ingre C., Roos P. M., Piehl F., Kamel F., Fang F. (2015). Risk factors for amyotrophic lateral sclerosis. *Clinical Epidemiology*.

[9] Lacorte E., Ferrigno L., Leoncini E., Corbo M., Boccia S., Vanacore N. (2016). Physical activity, and physical activity related to sports, leisure and occupational activity as risk factors for ALS: a systematic review. *Neuroscience and Biobehavioral Reviews*.

[10] Al-Chalabi A., Hardiman O. (2013). The epidemiology of ALS: a conspiracy of genes, environment and time. *Nature Reviews Neurology*.

[11] Yu B., Pamphlett R. (2017). Environmental insults: critical triggers for amyotrophic lateral sclerosis. *Translational Neurodegeneration*.

[12] Boylan K. (2015). Familial amyotrophic lateral sclerosis. *Neurologic Clinics*.

[13] DeJesus-Hernandez M., Mackenzie I. R., Boeve B. F. (2011). Expanded GGGGCC hexanucleotide repeat in noncoding region of C9ORF72 causes chromosome 9p-linked FTD and ALS. *Neuron*.

[14] Renton A. E., Majounie E., Waite A. (2011). A hexanucleotide repeat expansion in C9ORF72 is the cause of chromosome 9p21-linked ALS-FTD. *Neuron*.

[15] Deng H. X., Hentati A., Tainer J. A. (1993). Amyotrophic lateral sclerosis and structural defects in Cu,Zn superoxide dismutase. *Science*.

[16] Kaur S. J., McKeown S. R., Rashid S. (2016). Mutant SOD1 mediated pathogenesis of amyotrophic lateral sclerosis. *Gene*.

[17] Rosen D. R., Siddique T., Patterson D. (1993). Mutations in Cu/Zn superoxide dismutase gene are associated with familial amyotrophic lateral sclerosis. *Nature*.

[18] Synofzik M., Ronchi D., Keskin I. (2012). Mutant superoxide dismutase-1 indistinguishable from wild-type causes ALS. *Human Molecular Genetics*.

[19] Sreedharan J., Blair I. P., Tripathi V. B. (2008). TDP-43 mutations in familial and sporadic amyotrophic lateral sclerosis. *Science*.

[20] Kwiatkowski T. J., Bosco D. A., LeClerc A. L. (2009). Mutations in the FUS/TLS gene on chromosome 16 cause familial amyotrophic lateral sclerosis. *Science*.

[21] Vance C., Rogelj B., Hortobagyi T. (2009). Mutations in FUS, an RNA processing protein, cause familial amyotrophic lateral sclerosis type 6. *Science*.

[22] Oskarsson B., Gendron T. F., Staff N. P. (2018). Amyotrophic lateral sclerosis: an update for 2018. *Mayo Clinic Proceedings*.

[23] Bacman S. R., Bradley W. G., Moraes C. T. (2006). Mitochondrial involvement in amyotrophic lateral sclerosis: trigger or target?. *Molecular Neurobiology*.

[24] Lu H., Dong le W., Xie Y. Y., Wang X. P. (2016). Current therapy of drugs in amyotrophic lateral sclerosis. *Current Neuropharmacology*.

[25] Browne E. C., Abbott B. M. (2016). Recent progress towards an effective treatment of amyotrophic lateral sclerosis using the SOD1 mouse model in a preclinical setting. *European Journal of Medicinal Chemistry*.

[26] Pizzino G., Irrera N., Cucinotta M. (2017). Oxidative stress: harms and benefits for human health. *Oxidative Medicine and Cellular Longevity*.

[27] Brand M. D. (2010). The sites and topology of mitochondrial superoxide production. *Experimental Gerontology*.

[28] Kausar S., Wang F., Cui H. (2018). The role of mitochondria in reactive oxygen species generation and its implications for neurodegenerative diseases. *Cells*.

[29] Murphy M. P. (2009). How mitochondria produce reactive oxygen species. *The Biochemical Journal*.

[30] Starkov A. A., Fiskum G., Chinopoulos C. (2004). Mitochondrial alpha-ketoglutarate dehydrogenase complex generates reactive oxygen species. *The Journal of Neuroscience*.

[31] Tretter L., Adam-Vizi V. (2004). Generation of reactive oxygen species in the reaction catalyzed by -Ketoglutarate Dehydrogenase. *The Journal of Neuroscience*.

[32] Bunik V. I., Sievers C. (2002). Inactivation of the 2-oxo acid dehydrogenase complexes upon generation of intrinsic radical species. *European Journal of Biochemistry*.

[33] Orr A. L., Quinlan C. L., Perevoshchikova I. V., Brand M. D. (2012). A refined analysis of superoxide production by mitochondrial sn-glycerol 3-phosphate dehydrogenase. *The Journal of Biological Chemistry*.

[34] Muller F. L., Liu Y., Van Remmen H. (2004). Complex III releases superoxide to both sides of the inner mitochondrial membrane. *The Journal of Biological Chemistry*.

[35] Omura T. (2006). Mitochondrial P450s. *Chemico-Biological Interactions*.

[36] Forman H. J., Kennedy J. (1976). Dihydroorotate-dependent superoxide production in rat brain and liver. A function of the primary dehydrogenase. *Archives of Biochemistry and Biophysics*.

[37] Hey-Mogensen M., Goncalves R. L. S., Orr A. L., Brand M. D. (2014). Production of superoxide/H2O2 by dihydroorotate dehydrogenase in rat skeletal muscle mitochondria. *Free Radical Biology & Medicine*.

[38] Quinlan C. L., Orr A. L., Perevoshchikova I. V., Treberg J. R., Ackrell B. A., Brand M. D. (2012). Mitochondrial complex II can generate reactive oxygen species at high rates in both the forward and reverse reactions. *The Journal of Biological Chemistry*.

[39] Kaludercic N., Mialet-Perez J., Paolocci N., Parini A., di Lisa F. (2014). Monoamine oxidases as sources of oxidants in the heart. *Journal of Molecular and Cellular Cardiology*.

[40] Rani V., Deep G., Singh R. K., Palle K., Yadav U. C. S. (2016). Oxidative stress and metabolic disorders: pathogenesis and therapeutic strategies. *Life Sciences*.

[41] Birben E., Sahiner U. M., Sackesen C., Erzurum S., Kalayci O. (2012). Oxidative stress and antioxidant defense. *World Allergy Organization Journal*.

[42] Barber S. C., Mead R. J., Shaw P. J. (2006). Oxidative stress in ALS: a mechanism of neurodegeneration and a therapeutic target. *Biochimica et Biophysica Acta (BBA) - Molecular Basis of Disease*.

[43] Bodega G., Alique M., Puebla L., Carracedo J., Ramírez R. M. (2019). Microvesicles: ROS scavengers and ROS producers. *Journal of Extracellular Vesicles*.

[44] Phaniendra A., Jestadi D. B., Periyasamy L. (2015). Free radicals: properties, sources, targets, and their implication in various diseases. *Indian Journal of Clinical Biochemistry*.

[45] Niedzielska E., Smaga I., Gawlik M. (2016). Oxidative Stress in Neurodegenerative Diseases. *Molecular Neurobiology*.

[46] Tam O. H., Rozhkov N. V., Shaw R. (2019). Postmortem Cortex Samples Identify Distinct Molecular Subtypes of ALS: Retrotransposon Activation, Oxidative Stress, and Activated Glia. *Cell Reports*.

[47] Calingasan N. Y., Chen J., Kiaei M., Beal M. F. (2005). Beta-amyloid 42 accumulation in the lumbar spinal cord motor neurons of amyotrophic lateral sclerosis patients. *Neurobiology of Disease*.

[48] Ferrante R. J., Browne S. E., Shinobu L. A. (1997). Evidence of increased oxidative damage in both sporadic and familial amyotrophic lateral sclerosis. *Journal of Neurochemistry*.

[49] Shaw P. J., Ince P. G., Falkous G., Mantle D. (1995). Oxidative damage to protein in sporadic motor neuron disease spinal cord. *Annals of Neurology*.

[50] Simpson E. P., Henry Y. K., Henkel J. S., Smith R. G., Appel S. H. (2004). Increased lipid peroxidation in sera of ALS patients: a potential biomarker of disease burden. *Neurology*.

[51] Smith R. G., Henry Y. K., Mattson M. P., Appel S. H. (1998). Presence of 4-hydroxynonenal in cerebrospinal fluid of patients with sporadic amyotrophic lateral sclerosis. *Annals of Neurology*.

[52] Ihara Y., Nobukuni K., Takata H., Hayabara T. (2013). Oxidative stress and metal content in blood and cerebrospinal fluid of amyotrophic lateral sclerosis patients with and without a Cu, Zn-superoxide dismutase mutation. *Neurological Research*.

[53] Tohgi H., Abe T., Yamazaki K., Murata T., Ishizaki E., Isobe C. (1999). Remarkable increase in cerebrospinal fluid 3-nitrotyrosine in patients with sporadic amyotrophic lateral sclerosis. *Annals of Neurology*.

[54] Weishaupt J. H., Bartels C., Pölking E. (2006). Reduced oxidative damage in ALS by high-dose enteral melatonin treatment. *Journal of Pineal Research*.

[55] Mitsumoto H., Santella R. M., Liu X. (2009). Oxidative stress biomarkers in sporadic ALS. *Amyotrophic Lateral Sclerosis*.

[56] Agar J., Durham H. (2009). Relevance of oxidative injury in the pathogenesis of motor neuron diseases. *Amyotrophic Lateral Sclerosis and Other Motor Neuron Disorders*.

[57] Carrì M. T., Ferri A., Cozzolino M., Calabrese L., Rotilio G. (2003). Neurodegeneration in amyotrophic lateral sclerosis: the role of oxidative stress and altered homeostasis of metals. *Brain Research Bulletin*.

[58] Kraft A., Resch J., Johnson D., Johnson J. (2007). Activation of the Nrf2-ARE pathway in muscle and spinal cord during ALS-like pathology in mice expressing mutant SOD1. *Experimental Neurology*.

[59] Bowling A. C., Schulz J. B., Brown R. H., Beal M. F. (1993). Superoxide dismutase activity, oxidative damage, and mitochondrial energy metabolism in familial and sporadic amyotrophic lateral sclerosis. *Journal of Neurochemistry*.

[60] Niebroj-Dobosz I., Dziewulska D., Kwiecinski H. (2004). Oxidative damage to proteins in the spinal cord in amyotrophic lateral sclerosis ALS. *Folia Neuropathologica*.

[61] Shibata N., Nagai R., Uchida K. (2001). Morphological evidence for lipid peroxidation and protein glycoxidation in spinal cords from sporadic amyotrophic lateral sclerosis patients. *Brain Research*.

[62] Pedersen W. A., Fu W., Keller J. N. (1998). Protein modification by the lipid peroxidation product 4-hydroxynonenal in the spinal cords of amyotrophic lateral sclerosis patients. *Annals of Neurology*.

[63] Beal M. F., Ferrante R. J., Browne S. E., Matthews R. T., Kowall N. W., Brown R. H. (1997). Increased 3-nitrotyrosine in both sporadic and familial amyotrophic lateral sclerosis. *Annals of Neurology*.

[64] Abe K., Pan L.-H., Watanabe M., Konno H., Kato T., Itoyama Y. (2016). Upregulation of protein-tyrosine nitration in the anterior horn cells of amyotrophic lateral sclerosis. *Neurological Research*.

[65] Abe K., Pan L. H., Watanabe M., Kato T., Itoyama Y. (1995). Induction of nitrotyrosine-like immunoreactivity in the lower motor neuron of amyotrophic lateral sclerosis. *Neuroscience Letters*.

[66] Babu G. N., Kumar A., Chandra R. (2008). Oxidant-antioxidant imbalance in the erythrocytes of sporadic amyotrophic lateral sclerosis patients correlates with the progression of disease. *Neurochemistry International*.

[67] D’Amico E., Factor-Litvak P., Santella R. M., Mitsumoto H. (2013). Clinical perspective on oxidative stress in sporadic amyotrophic lateral sclerosis. *Free Radical Biology & Medicine*.

[68] Walczak J., Dębska-Vielhaber G., Vielhaber S. (2018). Distinction of sporadic and familial forms of ALS based on mitochondrial characteristics. *The FASEB Journal*.

[69] Ferri A., Cozzolino M., Crosio C. (2006). Familial ALS-superoxide dismutases associate with mitochondria and shift their redox potentials. *Proceedings of the National Academy of Sciences of the United States of America*.

[70] Chi L., Ke Y., Luo C., Gozal D., Liu R. (2007). Depletion of reduced glutathione enhances motor neuron degeneration in vitro and in vivo. *Neuroscience*.

[71] Pesaresi M. G., Amori I., Giorgi C. (2011). Mitochondrial redox signalling by p66Shc mediates ALS-like disease through Rac1 inactivation. *Human Molecular Genetics*.

[72] Pickles S., Destroismaisons L., Peyrard S. L. (2013). Mitochondrial damage revealed by immunoselection for ALS-linked misfolded SOD1. *Human Molecular Genetics*.

[73] Vargas M. R., Johnson D. A., Johnson J. A. (2011). Decreased glutathione accelerates neurological deficit and mitochondrial pathology in familial ALS-linked hSOD1(G93A) mice model. *Neurobiology of Disease*.

[74] Igoudjil A., Magrane J., Fischer L. R. (2011). In vivo pathogenic role of mutant SOD1 localized in the mitochondrial intermembrane space. *The Journal of Neuroscience*.

[75] McCord J. M., Fridovich I. (1969). Superoxide dismutase. An enzymic function for erythrocuprein (hemocuprein). *The Journal of Biological Chemistry*.

[76] Reddi A. R., Culotta V. C. (2013). SOD1 integrates signals from oxygen and glucose to repress respiration. *Cell*.

[77] Huai J., Zhang Z. (2019). Structural properties and interaction partners of familial ALS-associated SOD1 mutants. *Frontiers in Neurology*.

[78] Luquin N., Yu B., Trent R. J., Morahan J. M., Pamphlett R. (2008). An analysis of the entire SOD1 gene in sporadic ALS. *Neuromuscular Disorders*.

[79] Borchelt D. R., Lee M. K., Slunt H. S. (1994). Superoxide dismutase 1 with mutations linked to familial amyotrophic lateral sclerosis possesses significant activity. *Proceedings of the National Academy of Sciences of United States of America*.

[80] Yim M. B., Kang J. H., Yim H. S., Kwak H. S., Chock P. B., Stadtman E. R. (1996). A gain-of-function of an amyotrophic lateral sclerosis-associated Cu,Zn-superoxide dismutase mutant: An enhancement of free radical formation due to a decrease in Km for hydrogen peroxide. *Proceedings of the National Academy of Sciences of United States of America*.

[81] Kokić A. N., Stević Z., Stojanović S. (2013). Biotransformation of nitric oxide in the cerebrospinal fluid of amyotrophic lateral sclerosis patients. *Redox Report*.

[82] Reaume A. G., Elliott J. L., Hoffman E. K. (1996). Motor neurons in Cu/Zn superoxide dismutase-deficient mice develop normally but exhibit enhanced cell death after axonal injury. *Nature Genetics*.

[83] Ratovitski T., Corson L. B., Strain J. (1999). Variation in the biochemical/biophysical properties of mutant superoxide dismutase 1 enzymes and the rate of disease progression in familial amyotrophic lateral sclerosis kindreds. *Human Molecular Genetics*.

[84] Menzies F. M., Ince P. G., Shaw P. J. (2002). Mitochondrial involvement in amyotrophic lateral sclerosis. *Neurochemistry International*.

[85] Higgins C. M. J., Jung C., Ding H., Xu Z. (2002). Mutant Cu, Zn superoxide dismutase that causes motoneuron degeneration is present in mitochondria in the CNS. *The Journal of Neuroscience*.

[86] Liu D. (1996). The roles of free radicals in amyotrophic lateral sclerosis. *Journal of Molecular Neuroscience*.

[87] Valentine J. S., Doucette P. A., Zittin Potter S. (2005). Copper-zinc superoxide dismutase and amyotrophic lateral sclerosis. *Annual Review of Biochemistry*.

[88] Yim M. B., Chock P. B., Stadtman E. R. (1990). Copper, zinc superoxide dismutase catalyzes hydroxyl radical production from hydrogen peroxide. *Proceedings of the National Academy of Sciences of the United States of America*.

[89] Yim M. B., Chock P. B., Stadtman E. R. (1993). Enzyme function of copper, zinc superoxide dismutase as a free radical generator. *The Journal of Biological Chemistry*.

[90] Beckman J. S., Estévez A. G., Crow J. P., Barbeito L. (2001). Superoxide dismutase and the death of motoneurons in ALS. *Trends in Neurosciences*.

[91] Estévez A. G. (1999). Induction of Nitric Oxide -- Dependent Apoptosis in Motor Neurons by Zinc-Deficient Superoxide Dismutase. *Science*.

[92] Zhu C., Beck M. V., Griffith J. D., Deshmukh M., Dokholyan N. V. (2018). Large SOD1 aggregates, unlike trimeric SOD1, do not impact cell viability in a model of amyotrophic lateral sclerosis. *Proceedings of the National Academy of Sciences of the United States of America*.

[93] Chen X., Shang H., Qiu X. (2012). Oxidative modification of cysteine 111 promotes disulfide bond-independent aggregation of SOD1. *Neurochemical Research*.

[94] Ezzi S. A., Urushitani M., Julien J. P. (2007). Wild-type superoxide dismutase acquires binding and toxic properties of ALS-linked mutant forms through oxidation. *Journal of Neurochemistry*.

[95] Petrov D., Daura X., Zagrovic B. (2016). Effect of oxidative damage on the stability and dimerization of superoxide dismutase 1. *Biophysical Journal*.

[96] Neumann M., Sampathu D. M., Kwong L. K. (2006). Ubiquitinated TDP-43 in frontotemporal lobar degeneration and amyotrophic lateral sclerosis. *Science*.

[97] Pehar M., Beeson G., Beeson C. C., Johnson J. A., Vargas M. R. (2014). Mitochondria-targeted catalase reverts the neurotoxicity of hSOD1G93A astrocytes without extending the survival of ALS-linked mutant hSOD1 mice. *PLoS One*.

[98] Kato S., Kato M., Abe Y. (2005). Redox system expression in the motor neurons in amyotrophic lateral sclerosis (ALS): immunohistochemical studies on sporadic ALS, superoxide dismutase 1 (SOD1)-mutated familial ALS, and SOD1-mutated ALS animal models. *Acta Neuropathologica*.

[99] Killoy K. M., Harlan B. A., Pehar M., Helke K. L., Johnson J. A., Vargas M. R. (2018). Decreased glutathione levels cause overt motor neuron degeneration in hSOD1WT over-expressing mice. *Experimental Neurology*.

[100] Oka O. B. V., Bulleid N. J. (2013). Forming disulfides in the endoplasmic reticulum. *Biochimica et Biophysica Acta*.

[101] Espinosa-Diez C., Miguel V., Mennerich D. (2015). Antioxidant responses and cellular adjustments to oxidative stress. *Redox Biology*.

[102] Cozzolino M., Amori I., Pesaresi M. G., Ferri A., Nencini M., Carrì M. T. (2008). Cysteine 111 affects aggregation and cytotoxicity of mutant Cu,Zn-superoxide dismutase associated with familial amyotrophic lateral sclerosis. *The Journal of Biological Chemistry*.

[103] Niwa J., Yamada S. I., Ishigaki S. (2007). Disulfide bond mediates aggregation, toxicity, and ubiquitylation of familial amyotrophic lateral sclerosis-linked mutant SOD1. *The Journal of Biological Chemistry*.

[104] Valle C., Carri M. T. (2017). Cysteine Modifications in the Pathogenesis of ALS. *Frontiers in Molecular Neuroscience*.

[105] Thimmulappa R. K., Mai K. H., Srisuma S., Kensler T. W., Yamamoto M., Biswal S. (2002). Identification of Nrf2-regulated genes induced by the chemopreventive agent sulforaphane by oligonucleotide microarray. *Cancer Research*.

[106] Lee J. M., Calkins M. J., Chan K., Kan Y. W., Johnson J. A. (2003). Identification of the NF-E2-related factor-2-dependent genes conferring protection against oxidative stress in primary cortical astrocytes using oligonucleotide microarray analysis. *The Journal of Biological Chemistry*.

[107] Petri S., Korner S., Kiaei M. (2012). Nrf2/ARE signaling pathway: key mediator in oxidative stress and potential therapeutic target in ALS. *Neurology Research International*.

[108] Nguyen T., Nioi P., Pickett C. B. (2009). The Nrf2-antioxidant response element signaling pathway and its activation by oxidative stress. *The Journal of Biological Chemistry*.

[109] Nguyen T., Sherratt P. J., Pickett C. B. (2003). Regulatory mechanisms controlling gene expression mediated by the antioxidant response element. *Annual Review of Pharmacology and Toxicology*.

[110] Kirby J., Halligan E., Baptista M. J. (2005). Mutant SOD1 alters the motor neuronal transcriptome: implications for familial ALS. *Brain*.

[111] Sarlette A., Krampfl K., Grothe C., Neuhoff N. ., Dengler R., Petri S. (2008). Nuclear erythroid 2-related factor 2-antioxidative response element signaling pathway in motor cortex and spinal cord in amyotrophic lateral sclerosis. *Journal of Neuropathology and Experimental Neurology*.

[112] Wu D. C., Re D. B., Nagai M., Ischiropoulos H., Przedborski S. (2006). The inflammatory NADPH oxidase enzyme modulates motor neuron degeneration in amyotrophic lateral sclerosis mice. *Proceedings of the National Academy of Sciences of the United States of America*.

[113] Marden J. J., Harraz M. M., Williams A. J. (2007). Redox modifier genes in amyotrophic lateral sclerosis in mice. *The Journal of Clinical Investigation*.

[114] Harraz M. M., Marden J. J., Zhou W. (2008). SOD1 mutations disrupt redox-sensitive Rac regulation of NADPH oxidase in a familial ALS model. *Journal of Clinical Investigation*.

[115] Tian Y. P., Che F. Y., Su Q. P. (2017). Effects of mutant TDP-43 on the Nrf2/ARE pathway and protein expression of MafK and JDP2 in NSC-34 cells. *Genetics and Molecular Research*.

[116] Moujalled D., Grubman A., Acevedo K. (2017). TDP-43 mutations causing amyotrophic lateral sclerosis are associated with altered expression of RNA-binding protein hnRNP K and affect the Nrf2 antioxidant pathway. *Human Molecular Genetics*.

[117] Duan W., Li X., Shi J., Guo Y., Li Z., Li C. (2010). Mutant TAR DNA-binding protein-43 induces oxidative injury in motor neuron-like cell. *Neuroscience*.

[118] Hergesheimer R. C., Chami A. A., de Assis D. R. (2019). The debated toxic role of aggregated TDP-43 in amyotrophic lateral sclerosis: a resolution in sight?. *Brain*.

[119] François-Moutal L., Perez-Miller S., Scott D. D., Miranda V. G., Mollasalehi N., Khanna M. (2019). Structural insights into TDP-43 and effects of post-translational modifications. *Frontiers in Molecular Neuroscience*.

[120] Cohen T. J., Hwang A. W., Unger T., Trojanowski J. Q., Lee V. M. Y. (2012). Redox signalling directly regulates TDP-43 via cysteine oxidation and disulphide cross-linking. *The EMBO Journal*.

[121] Chang C. K., Chiang M. H., Toh E. K. W., Chang C. F., Huang T. H. (2013). Molecular mechanism of oxidation-induced TDP-43 RRM1 aggregation and loss of function. *FEBS Letters*.

[122] Vance C., Scotter E. L., Nishimura A. L. (2013). ALS mutant FUS disrupts nuclear localization and sequesters wild-type FUS within cytoplasmic stress granules. *Human Molecular Genetics*.

[123] Deng Q., Holler C. J., Taylor G. (2014). FUS is phosphorylated by DNA-PK and accumulates in the cytoplasm after DNA damage. *The Journal of Neuroscience*.

[124] Wang H., Guo W., Mitra J. (2018). Mutant FUS causes DNA ligation defects to inhibit oxidative damage repair in amyotrophic lateral sclerosis. *Nature Communications*.

[125] Lai J. D., Ichida J. K. (2019). C9ORF72 protein function and immune dysregulation in amyotrophic lateral sclerosis. *Neuroscience Letters*.

[126] Mori K., Arzberger T., Grässer F. A. (2013). Bidirectional transcripts of the expanded C9orf72 hexanucleotide repeat are translated into aggregating dipeptide repeat proteins. *Acta Neuropathologica*.

[127] Mori K., Weng S. M., Arzberger T. (2013). The C9orf72 GGGGCC repeat is translated into aggregating dipeptide-repeat proteins in FTLD/ALS. *Science*.

[128] Lopez-Gonzalez R., Lu Y., Gendron T. F. (2016). Poly(GR) in C9ORF72-related ALS/FTD compromises mitochondrial function and increases oxidative stress and DNA damage in iPSC-derived motor neurons. *Neuron*.

[129] Birger A., Ben-Dor I., Ottolenghi M. (2019). Human iPSC-derived astrocytes from ALS patients with mutated C9ORF72 show increased oxidative stress and neurotoxicity. *eBioMedicine*.

[130] Ghasemi M., Brown R. H. (2018). Genetics of amyotrophic lateral sclerosis. *Cold Spring Harbor Perspectives in Medicine*.

[131] Cho G. W., Kang B. Y., Kim S. H. (2010). Human angiogenin presents neuroprotective and migration effects in neuroblastoma cells. *Molecular and Cellular Biochemistry*.

[132] Fernández-Santiago R., Hoenig S., Lichtner P. (2009). Identification of novel angiogenin (ANG) gene missense variants in German patients with amyotrophic lateral sclerosis. *Journal of Neurology*.

[133] Hoang T. T., Johnson D. A., Raines R. T., Johnson J. A. (2019). Angiogenin activates the astrocytic Nrf2/antioxidant-response element pathway and thereby protects murine neurons from oxidative stress. *The Journal of Biological Chemistry*.

[134] Wills A. M., Cronin S., Slowik A. (2009). A large-scale international meta-analysis of paraoxonase gene polymorphisms in sporadic ALS. *Neurology*.

[135] Valdmanis P. N., Kabashi E., Dyck A. (2008). Association of paraoxonase gene cluster polymorphisms with ALS in France, Quebec, and Sweden. *Neurology*.

[136] Saeed M., Siddique N., Hung W. Y. (2006). Paraoxonase cluster polymorphisms are associated with sporadic ALS. *Neurology*.

[137] Ng C. J., Wadleigh D. J., Gangopadhyay A. (2001). Paraoxonase-2 is a ubiquitously expressed protein with antioxidant properties and is capable of preventing cell-mediated oxidative modification of low density lipoprotein. *The Journal of Biological Chemistry*.

[138] Zoccolella S., Santamato A., Lamberti P. (2009). Current and emerging treatments for amyotrophic lateral sclerosis. *Neuropsychiatric Disease and Treatment*.

[139] Bedlack R. S., Traynor B. J., Cudkowicz M. E. (2007). Emerging disease-modifying therapies for the treatment of motor neuron disease/amyotropic lateral sclerosis. *Expert Opinion on Emerging Drugs*.

[140] Gurney M. E., Cutting F. B., Zhai P. (1996). Benefit of vitamin E, riluzole, and gababapentin in a transgenic model of familial amyotrophic lateral sclerosis. *Annals of Neurology*.

[141] Michal Freedman D., Kuncl R. W., Weinstein S. J., Malila N., Virtamo J., Albanes D. (2012). Vitamin E serum levels and controlled supplementation and risk of amyotrophic lateral sclerosis. *Amyotrophic Lateral Sclerosis and Frontotemporal Degeneration*.

[142] de Bustos F., Jiménez-Jiménez F. J., Molina J. A. (1998). Cerebrospinal fluid levels of alpha-tocopherol in amyotrophic lateral sclerosis. *Journal of Neural Transmission*.

[143] Iwasaki Y., Ikeda K., Kinoshita M. (1995). Vitamin A and E levels are normal in amyotrophic lateral sclerosis. *Journal of the Neurological Sciences*.

[144] Wang H., O'Reilly E. J., Weisskopf M. G. (2011). Vitamin E intake and risk of amyotrophic lateral sclerosis: a pooled analysis of data from 5 prospective cohort studies. *American Journal of Epidemiology*.

[145] Veldink J. H., Kalmijn S., Groeneveld G.-J. (2006). Intake of polyunsaturated fatty acids and vitamin E reduces the risk of developing amyotrophic lateral sclerosis. *Journal of Neurology, Neurosurgery, and Psychiatry*.

[146] Ascherio A., Weisskopf M. G., O'Reilly E. J. (2005). Vitamin E intake and risk of amyotrophic lateral sclerosis. *Annals of Neurology*.

[147] Desnuelle C., Dib M., Garrel C., Favier A. (2009). A double-blind, placebo-controlled randomized clinical trial of alpha-tocopherol (vitamin E) in the treatment of amyotrophic lateral sclerosis. ALS riluzole-tocopherol study group. *Amyotrophic Lateral Sclerosis and Other Motor Neuron Disorders*.

[148] Galbussera A., Tremolizzo L., Brighina L. (2006). Vitamin E intake and quality of life in amyotrophic lateral sclerosis patients: a follow-up case series study. *Neurological Sciences*.

[149] Graf M., Ecker D., Horowski R. (2005). High dose vitamin E therapy in amyotrophic lateral sclerosis as add-on therapy to riluzole: results of a placebo-controlled double-blind study. *Journal of Neural Transmission (Vienna)*.

[150] Pappert E. J., Tangney C. C., Goetz C. G. (1996). Alpha-tocopherol in the ventricular cerebrospinal fluid of Parkinson's disease patients: dose-response study and correlations with plasma levels. *Neurology*.

[151] Kraus R. L., Pasieczny R., Lariosa-Willingham K., Turner M. S., Jiang A., Trauger J. W. (2005). Antioxidant properties of minocycline: neuroprotection in an oxidative stress assay and direct radical-scavenging activity. *Journal of Neurochemistry*.

[152] Baldinger R., Katzberg H. D., Weber M. (2012). Treatment for cramps in amyotrophic lateral sclerosis/motor neuron disease. *Cochrane Database of Systematic Reviews*.

[153] Andreassen O. A., Dedeoglu A., Klivenyi P., Beal M. F., Bush A. I. (2000). N-acetyl-L-cysteine improves survival and preserves motor performance in an animal model of familial amyotrophic lateral sclerosis. *Neuroreport*.

[154] Burgunder J. M., Varriale A., Lauterburg B. H. (1989). Effect of N-acetylcysteine on plasma cysteine and glutathione following paracetamol administration. *European Journal of Clinical Pharmacology*.

[155] Beretta S., Sala G., Mattavelli L. (2003). Mitochondrial dysfunction due to mutant copper/zinc superoxide dismutase associated with amyotrophic lateral sclerosis is reversed by N-acetylcysteine. *Neurobiology of Disease*.

[156] Louwerse E. S., Weverling G. J., Bossuyt P. M. M., Meyjes F. E. P., de Jong J. M. B. V. (1995). Randomized, double-blind, controlled trial of acetylcysteine in amyotrophic lateral sclerosis. *Archives of Neurology*.

[157] Bresolin N., Bet L., Binda A. (1988). Clinical and biochemical correlations in mitochondrial myopathies treated with coenzyme Q10. *Neurology*.

[158] Do T. Q., Schultz J. R., Clarke C. F. (1996). Enhanced sensitivity of ubiquinone-deficient mutants of Saccharomyces cerevisiae to products of autoxidized polyunsaturated fatty acids. *Proceedings of the National Academy of Sciences of the United States of America*.

[159] Matthews R. T., Yang L., Browne S., Baik M., Beal M. F. (1998). Coenzyme Q10 administration increases brain mitochondrial concentrations and exerts neuroprotective effects. *Proceedings of the National Academy of Sciences of the United States of America*.

[160] Lucchetti J., Marino M., Papa S. (2013). A mouse model of familial ALS has increased CNS levels of endogenous ubiquinol9/10 and does not benefit from exogenous administration of ubiquinol10. *PLoS One*.

[161] Sohmiya M., Tanaka M., Suzuki Y., Tanino Y., Okamoto K., Yamamoto Y. (2005). An increase of oxidized coenzyme Q-10 occurs in the plasma of sporadic ALS patients. *Journal of the Neurological Sciences*.

[162] Molina J. A., de Bustos F., Jiménez-Jiménez F. J. (2000). Serum levels of coenzyme Q10 in patients with amyotrophic lateral sclerosis. *Journal of Neural Transmission (Vienna)*.

[163] Ferrante K. L., Shefner J., Zhang H. (2005). Tolerance of high-dose (3,000 mg/day) coenzyme Q10 in ALS. *Neurology*.

[164] Levy G., Kaufmann P., Buchsbaum R. (2006). A two-stage design for a phase II clinical trial of coenzyme Q10 in ALS. *Neurology*.

[165] Kaufmann P., Thompson J. L. P., Levy G. (2009). Phase II trial of CoQ10 for ALS finds insufficient evidence to justify phase III. *Annals of Neurology*.

[166] Neves Carvalho A., Firuzi O., Joao Gama M., van Horssen J., Saso L. (2017). Oxidative stress and antioxidants in neurological diseases: is there still hope?. *Current Drug Targets*.

[167] Vargas M. R., Johnson D. A., Sirkis D. W., Messing A., Johnson J. A. (2008). Nrf2 activation in astrocytes protects against neurodegeneration in mouse models of familial amyotrophic lateral sclerosis. *The Journal of Neuroscience*.

[168] Guo Y., Zhang Y., Wen D. (2013). The modest impact of transcription factor Nrf2 on the course of disease in an ALS animal model. *Laboratory Investigation*.

[169] van Muiswinkel F., Kuiperij H. (2005). The Nrf2-ARE signalling pathway: promising drug target to combat oxidative stress in neurodegenerative disorders. *Current Drug Targets. CNS and Neurological Disorders*.

[170] Tanaka K., Kanno T., Yanagisawa Y. (2014). A novel acylaminoimidazole derivative, WN1316, alleviates disease progression via suppression of glial inflammation in ALS mouse model. *PLoS One*.

[171] Jiang H., Tian X., Guo Y., Duan W., Bu H., Li C. (2011). Activation of nuclear factor erythroid 2-related factor 2 cytoprotective signaling by curcumin protect primary spinal cord astrocytes against oxidative toxicity. *Biological & Pharmaceutical Bulletin*.

[172] Bhatia N. K., Srivastava A., Katyal N. (2015). Curcumin binds to the pre-fibrillar aggregates of Cu/Zn superoxide dismutase (SOD1) and alters its amyloidogenic pathway resulting in reduced cytotoxicity. *Biochimica et Biophysica Acta*.

[173] Lu J., Duan W., Guo Y. (2012). Mitochondrial dysfunction in human TDP-43 transfected NSC34 cell lines and the protective effect of dimethoxy curcumin. *Brain Research Bulletin*.

[174] Ahmadi M., Agah E., Nafissi S. (2018). Safety and efficacy of nanocurcumin as add-on therapy to riluzole in patients with amyotrophic lateral sclerosis: a pilot randomized clinical trial. *Neurotherapeutics*.

[175] Chico L., Ienco E. C., Bisordi C. (2018). Amyotrophic lateral sclerosis and oxidative stress: a double-blind therapeutic trial after curcumin supplementation. *CNS & Neurological Disorders Drug Targets*.

[176] Rakotoarisoa M., Angelova A. (2018). Amphiphilic nanocarrier systems for curcumin delivery in neurodegenerative disorders. *Medicines*.

[177] Tripodo G., Chlapanidas T., Perteghella S. (2015). Mesenchymal stromal cells loading curcumin-INVITE-micelles: a drug delivery system for neurodegenerative diseases. *Colloids and Surfaces, B: Biointerfaces*.

[178] Neymotin A., Calingasan N. Y., Wille E. (2011). Neuroprotective effect of Nrf2/ARE activators, CDDO ethylamide and CDDO trifluoroethylamide, in a mouse model of amyotrophic lateral sclerosis. *Free Radical Biology & Medicine*.

[179] Mead R. J., Higginbottom A., Allen S. P. (2013). *S*[+] Apomorphine is a CNS penetrating activator of the Nrf2-ARE pathway with activity in mouse and patient fibroblast models of amyotrophic lateral sclerosis. *Free Radical Biology & Medicine*.

[180] Na H. K., Surh Y. J. (2008). Modulation of Nrf2-mediated antioxidant and detoxifying enzyme induction by the green tea polyphenol EGCG. *Food and Chemical Toxicology*.

[181] Pervin M., Unno K., Nakagawa A. (2017). Blood brain barrier permeability of (-)-epigallocatechin gallate, its proliferation-enhancing activity of human neuroblastoma SH-SY5Y cells, and its preventive effect on age-related cognitive dysfunction in mice. *Biochemistry and Biophysics Reports*.

[182] Koh S. H., Kwon H., Kim K. S. (2004). Epigallocatechin gallate prevents oxidative-stress-induced death of mutant Cu/Zn-superoxide dismutase (G93A) motoneuron cells by alteration of cell survival and death signals. *Toxicology*.

[183] Koh S. H., Lee S. M., Kim H. Y. (2006). The effect of epigallocatechin gallate on suppressing disease progression of ALS model mice. *Neuroscience Letters*.

[184] Xu Z., Chen S., Li X., Luo G., Li L., le W. (2006). Neuroprotective effects of (-)-epigallocatechin-3-gallate in a transgenic mouse model of amyotrophic lateral sclerosis. *Neurochemical Research*.

[185] Pattee G. L., Post G. R., Gerber R. E., Bennett, Jr J. P. (2009). Reduction of oxidative stress in amyotrophic lateral sclerosis following pramipexole treatment. *Amyotrophic Lateral Sclerosis and Other Motor Neuron Disorders*.

[186] Cudkowicz M., Bozik M. E., Ingersoll E. W. (2011). The effects of dexpramipexole (KNS-760704) in individuals with amyotrophic lateral sclerosis. *Nature Medicine*.

[187] Danzeisen R., Schwalenstoecker B., Gillardon F. (2005). Targeted antioxidative and neuroprotective properties of the dopamine agonist pramipexole and its nondopaminergic enantiomer SND919CL2x [(+)2-amino-4,5,6,7-tetrahydro-6-Lpropylamino-benzathiazole dihydrochloride]. *The Journal of Pharmacology and Experimental Therapeutics*.

[188] Ferrari-Toninelli G., Maccarinelli G., Uberti D., Buerger E., Memo M. (2010). Mitochondria-targeted antioxidant effects of S(-) and R(+) pramipexole. *BMC Pharmacology*.

[189] Abramova N. A., Cassarino D. S., Khan S. M., Painter T. W., Bennett J. P. (2002). Inhibition by R(+) or S(-) pramipexole of caspase activation and cell death induced by methylpyridinium ion or beta amyloid peptide in SH-SY5Y neuroblastoma. *Journal of Neuroscience Research*.

[190] Bozik M. E., Mather J. L., Kramer W. G., Gribkoff V. K., Ingersoll E. W. (2011). Safety, tolerability, and pharmacokinetics of KNS-760704 (dexpramipexole) in healthy adult subjects. *Journal of Clinical Pharmacology*.

[191] Rudnicki S. A., Berry J. D., Ingersoll E. (2013). Dexpramipexole effects on functional decline and survival in subjects with amyotrophic lateral sclerosis in a phase II study: subgroup analysis of demographic and clinical characteristics. *Amyotrophic Lateral Sclerosis and Frontotemporal Degeneration*.

[192] Cudkowicz M. E., van den Berg L. H., Shefner J. M. (2013). Dexpramipexole versus placebo for patients with amyotrophic lateral sclerosis (EMPOWER): a randomised, double-blind, phase 3 trial. *Lancet Neurology*.

[193] Vieira F. G., LaDow E., Moreno A. (2014). Dexpramipexole is ineffective in two models of ALS related neurodegeneration. *PLoS One*.

[194] Tan D. X., Reiter R., Manchester L. (2002). Chemical and physical properties and potential mechanisms: melatonin as a broad spectrum antioxidant and free radical scavenger. *Current Topics in Medicinal Chemistry*.

[195] Ganie S. A., Dar T. A., Bhat A. H. (2016). Melatonin: a potential anti-oxidant therapeutic agent for mitochondrial dysfunctions and related disorders. *Rejuvenation Research*.

[196] Pandi-Perumal S. R., BaHammam A. S., Brown G. M. (2013). Melatonin antioxidative defense: therapeutical implications for aging and neurodegenerative processes. *Neurotoxicity Research*.

[197] Leon J., Acuna-Castroviejo D., Escames G., Tan D. X., Reiter R. J. (2005). Melatonin mitigates mitochondrial malfunction. *Journal of Pineal Research*.

[198] Zhang Y., Cook A., Kim J. (2013). Melatonin inhibits the caspase-1/cytochrome c/caspase-3 cell death pathway, inhibits MT1 receptor loss and delays disease progression in a mouse model of amyotrophic lateral sclerosis. *Neurobiology of Disease*.

[199] Dardiotis E., Panayiotou E., Feldman M. L. (2013). Intraperitoneal melatonin is not neuroprotective in the G93ASOD1 transgenic mouse model of familial ALS and may exacerbate neurodegeneration. *Neuroscience Letters*.

[200] Barua S., Kim J. Y., Yenari M. A., Lee J. E. (2019). The role of NOX inhibitors in neurodegenerative diseases. *IBRO Reports*.

[201] Saso L., Firuzi O. (2014). Pharmacological applications of antioxidants: lights and shadows. *Current Drug Targets*.

[202] Sun A. Y., Wang Q., Simonyi A., Sun G. Y. (2008). Botanical phenolics and brain health. *Neuromolecular Medicine*.

[203] Marchetto M. C., Muotri A. R., Mu Y., Smith A. M., Cezar G. G., Gage F. H. (2008). Non-cell-autonomous effect of human SOD1 G37R astrocytes on motor neurons derived from human embryonic stem cells. *Cell Stem Cell*.

[204] Heumüller S., Wind S., Barbosa-Sicard E. (2008). Apocynin is not an inhibitor of vascular NADPH oxidases but an antioxidant. *Hypertension*.

[205] Trumbull K. A., McAllister D., Gandelman M. M. (2012). Diapocynin and apocynin administration fails to significantly extend survival in G93A SOD1 ALS mice. *Neurobiology of Disease*.

[206] Stefanska J., Sokolowska M., Sarniak A. (2010). Apocynin decreases hydrogen peroxide and nitrate concentrations in exhaled breath in healthy subjects. *Pulmonary Pharmacology & Therapeutics*.

[207] Dikalov S. (2011). Cross talk between mitochondria and NADPH oxidases. *Free Radical Biology & Medicine*.

[208] Zhang X. R., Zhou W. X., Zhang Y. X. (2018). Improvements in SOD mimic AEOL-10150, a potent broad-spectrum antioxidant. *Military Medical Research*.

[209] Patel M., Day B. J. (1999). Metalloporphyrin class of therapeutic catalytic antioxidants. *Trends in Pharmacological Sciences*.

[210] Petri S., Kiaei M., Kipiani K. (2006). Additive neuroprotective effects of a histone deacetylase inhibitor and a catalytic antioxidant in a transgenic mouse model of amyotrophic lateral sclerosis. *Neurobiology of Disease*.

[211] Crow J. P., Calingasan N. Y., Chen J., Hill J. L., Beal M. F. (2005). Manganese porphyrin given at symptom onset markedly extends survival of ALS mice. *Annals of Neurology*.

[212] Shichinohe H., Kuroda S., Yasuda H. (2004). Neuroprotective effects of the free radical scavenger Edaravone (MCI-186) in mice permanent focal brain ischemia. *Brain Research*.

[213] Abe K., Itoyama Y., Sobue G. (2014). Confirmatory double-blind, parallel-group, placebo-controlled study of efficacy and safety of edaravone (MCI-186) in amyotrophic lateral sclerosis patients. *Amyotrophic Lateral Sclerosis and Frontotemporal Degeneration*.

[214] Firuzi O., Miri R., Tavakkoli M., Saso L. (2011). Antioxidant therapy: current status and future prospects. *Current Medicinal Chemistry*.

[215] Mizuno A., Umemura K., Nakashima M. (1998). Inhibitory effect of MCI-186, a free radical scavenger, on cerebral ischemia following rat middle cerebral artery occlusion. *General Pharmacology*.

[216] Yamamoto T., Yuki S., Watanabe T., Mitsuka M., Saito K. I., Kogure K. (1997). Delayed neuronal death prevented by inhibition of increased hydroxyl radical formation in a transient cerebral ischemia. *Brain Research*.

[217] Takayasu Y., Nakaki J., Kawasaki T. (2007). Edaravone, a radical scavenger, inhibits mitochondrial permeability transition pore in rat brain. *Journal of Pharmacological Sciences*.

[218] Jami M. S., Salehi-Najafabadi Z., Ahmadinejad F. (2015). Edaravone leads to proteome changes indicative of neuronal cell protection in response to oxidative stress. *Neurochemistry International*.

[219] Zhang M., Teng C. H., Wu F. F. (2019). Edaravone attenuates traumatic brain injury through anti-inflammatory and anti-oxidative modulation. *Experimental and Therapeutic Medicine*.

[220] Banno M., Mizuno T., Kato H. (2005). The radical scavenger edaravone prevents oxidative neurotoxicity induced by peroxynitrite and activated microglia. *Neuropharmacology*.

[221] Wang Q., Zhang X., Chen S. (2011). Prevention of motor neuron degeneration by novel iron chelators in SOD1<sup>G93A</sup> transgenic mice of amyotrophic lateral sclerosis. *Neurodegenerative Diseases*.

[222] Miyamoto N., Maki T., Pham L. D. D. (2013). Oxidative stress interferes with white matter renewal after prolonged cerebral hypoperfusion in mice. *Stroke*.

[223] Aoki M., Warita H., Mizuno H., Suzuki N., Yuki S., Itoyama Y. (2011). Feasibility study for functional test battery of SOD transgenic rat (H46R) and evaluation of edaravone, a free radical scavenger. *Brain Research*.

[224] Ito H., Wate R., Zhang J. (2008). Treatment with edaravone, initiated at symptom onset, slows motor decline and decreases SOD1 deposition in ALS mice. *Experimental Neurology*.

[225] Yoshino H., Kimura A. (2006). Investigation of the therapeutic effects of edaravone, a free radical scavenger, on amyotrophic lateral sclerosis (Phase II study). *Amyotrophic Lateral Sclerosis*.

[226] THE WRITING GROUP ON BEHALF OF THE EDARAVONE (MCI-186) ALS 19 STUDY GROUP (2017). Open-label 24-week extension study of edaravone (MCI-186) in amyotrophic lateral sclerosis. *Amyotrophic Lateral Sclerosis and Frontotemporal Degeneration*.

[227] Hardiman O., van den Berg L. H. (2017). Edaravone: a new treatment for ALS on the horizon?. *Lancet Neurology*.

[228] Bensimon G., Lacomblez L., Meininger V. (1994). A controlled trial of riluzole in amyotrophic lateral sclerosis. ALS/Riluzole Study Group. *The New England Journal of Medicine*.

[229] Miller R. G., Bouchard J. P., Duquette P. (1996). Clinical trials of riluzole in patients with ALS. *Neurology*.

[230] Jackson M., Lladó J., Rothstein J. D. (2005). Therapeutic developments in the treatment of amyotrophic lateral sclerosis. *Expert Opinion on Investigational Drugs*.

[231] Jaiswal M. K. (2019). Riluzole and edaravone: a tale of two amyotrophic lateral sclerosis drugs. *Medicinal Research Reviews*.

[232] Rego A. C., Oliveira C. R. (2003). Mitochondrial dysfunction and reactive oxygen species in excitotoxicity and apoptosis: implications for the pathogenesis of neurodegenerative diseases. *Neurochemical Research*.

[233] Noh K. M., Hwang J. Y., Shin H. C., Koh J. Y. (2000). A novel neuroprotective mechanism of riluzole: direct inhibition of protein kinase C. *Neurobiology of Disease*.

[234] Koh J. Y., Kim D. K., Hwang J. Y., Kim Y. H., Seo J. H. (1999). Antioxidative and proapoptotic effects of riluzole on cultured cortical neurons. *Journal of Neurochemistry*.

[235] Deng Y., Xu Z.-F., Liu W., Xu B., Yang H.-B., Wei Y.-G. (2012). Riluzole-triggered GSH synthesis via activation of glutamate transporters to antagonize methylmercury-induced oxidative stress in rat cerebral cortex. *Oxidative Medicine and Cellular Longevity*.

[236] Sala G., Arosio A., Conti E. (2019). Riluzole selective antioxidant effects in cell models expressing amyotrophic lateral sclerosis endophenotypes. *Clinical Psychopharmacology and Neuroscience*.

[237] Hogg M. C., Halang L., Woods I., Coughlan K. S., PREHN J. H. M. (2018). Riluzole does not improve lifespan or motor function in three ALS mouse models. *Amyotrophic Lateral Sclerosis and Frontotemporal Degeneration*.

[238] Nagase M., Yamamoto Y., Miyazaki Y., Yoshino H. (2016). Increased oxidative stress in patients with amyotrophic lateral sclerosis and the effect of edaravone administration. *Redox Report*.

[239] Yaku K., Okabe K., Nakagawa T. (2018). NAD metabolism: implications in aging and longevity. *Ageing Research Reviews*.

[240] Salminen A., Kaarniranta K., Kauppinen A. (2013). Crosstalk between oxidative stress and SIRT1: impact on the aging process. *International Journal of Molecular Sciences*.

[241] Austin S., St-Pierre J. (2012). PGC1 and mitochondrial metabolism - emerging concepts and relevance in ageing and neurodegenerative disorders. *Journal of Cell Science*.

[242] Fernandez-Marcos P. J., Auwerx J. (2011). Regulation of PGC-1*α*, a nodal regulator of mitochondrial biogenesis. *The American Journal of Clinical Nutrition*.

[243] Pasinetti G. M., Bilski A. E., Zhao W. (2013). Sirtuins as therapeutic targets of ALS. *Cell Research*.

[244] Körner S., Böselt S., Thau N., Rath K. J., Dengler R., Petri S. (2013). Differential sirtuin expression patterns in amyotrophic lateral sclerosis (ALS) postmortem tissue: neuroprotective or neurotoxic properties of sirtuins in ALS?. *Neurodegenerative Diseases*.

[245] Han S., Choi J. R., Soon Shin K., Kang S. J. (2012). Resveratrol upregulated heat shock proteins and extended the survival of G93A-SOD1 mice. *Brain Research*.

[246] Song W., Song Y., Kincaid B., Bossy B., Bossy-Wetzel E. (2013). Mutant SOD1G93A triggers mitochondrial fragmentation in spinal cord motor neurons: neuroprotection by SIRT3 and PGC-1*α*. *Neurobiology of Disease*.

[247] Braidy N., Berg J., Clement J. (2019). Role of nicotinamide adenine dinucleotide and related precursors as therapeutic targets for age-related degenerative diseases: rationale, biochemistry, pharmacokinetics, and outcomes. *Antioxidants & Redox Signaling*.

[248] Harlan B. A., Pehar M., Sharma D. R., Beeson G., Beeson C. C., Vargas M. R. (2016). Enhancing NAD+ salvage pathway reverts the toxicity of primary astrocytes expressing amyotrophic lateral sclerosis-linked mutant superoxide dismutase 1 (SOD1). *The Journal of Biological Chemistry*.

[249] Harlan B. A., Killoy K. M., Pehar M., Liu L., Auwerx J., Vargas M. R. (2020). Evaluation of the NAD(+) biosynthetic pathway in ALS patients and effect of modulating NAD(+) levels in hSOD1-linked ALS mouse models. *Experimental Neurology*.

[250] Ma J., Feng B., Kong D. (2020). Production and validation of human induced pluripotent stem cell line from sporadic amyotrophic lateral sclerosis (SALS). *Stem Cell Research*.

[251] Zhou Q., Zhu L., Qiu W. (2020). Nicotinamide riboside enhances mitochondrial proteostasis and adult neurogenesis through activation of mitochondrial unfolded protein response signaling in the brain of ALS SOD1G93AMice. *International Journal of Biological Sciences*.

[252] Harlan B. A., Pehar M., Killoy K. M., Vargas M. R. (2019). Enhanced SIRT6 activity abrogates the neurotoxic phenotype of astrocytes expressing ALS-linked mutant SOD1. *The FASEB Journal*.

[253] de la Rubia J. E., Drehmer E., Platero J. É. L. (2019). Efficacy and tolerability of EH301 for amyotrophic lateral sclerosis: a randomized, double-blind, placebo-controlled human pilot study. *Amyotrophic Lateral Sclerosis and Frontotemporal Degeneration*.

[254] Murphy M. P., Smith R. A. (2007). Targeting antioxidants to mitochondria by conjugation to lipophilic cations. *Annual Review of Pharmacology and Toxicology*.

[255] Finichiu P. G., James A. M., Larsen L., Smith R. A. J., Murphy M. P. (2013). Mitochondrial accumulation of a lipophilic cation conjugated to an ionisable group depends on membrane potential, pH gradient and pK(a): implications for the design of mitochondrial probes and therapies. *Journal of Bioenergetics and Biomembranes*.

[256] Kelso G. F., Porteous C. M., Coulter C. V. (2001). Selective targeting of a redox-active ubiquinone to mitochondria within cells: antioxidant and antiapoptotic properties. *The Journal of Biological Chemistry*.

[257] Cochemé H. M., Kelso G. F., James A. M. (2007). Mitochondrial targeting of quinones: therapeutic implications. *Mitochondrion*.

[258] Smith R. A., Murphy M. P. (2010). Animal and human studies with the mitochondria-targeted antioxidant MitoQ. *Annals of the New York Academy of Sciences*.

[259] Solesio M. E., Prime T. A., Logan A. (2013). The mitochondria-targeted anti-oxidant MitoQ reduces aspects of mitochondrial fission in the 6-OHDA cell model of Parkinson's disease. *Biochimica et Biophysica Acta*.

[260] Ghosh A., Chandran K., Kalivendi S. V. (2010). Neuroprotection by a mitochondria-targeted drug in a Parkinson's disease model. *Free Radical Biology & Medicine*.

[261] McManus M. J., Murphy M. P., Franklin J. L. (2011). The mitochondria-targeted antioxidant MitoQ prevents loss of spatial memory retention and early neuropathology in a transgenic mouse model of Alzheimer's disease. *The Journal of Neuroscience*.

[262] Manczak M., Mao P., Calkins M. J. (2010). Mitochondria-targeted antioxidants protect against amyloid-beta toxicity in Alzheimer's disease neurons. *Journal of Alzheimer's Disease*.

[263] Snow B. J., Rolfe F. L., Lockhart M. M. (2010). A double-blind, placebo-controlled study to assess the mitochondria-targeted antioxidant MitoQ as a disease-modifying therapy in Parkinson's disease. *Movement Disorders*.

[264] Cassina P., Cassina A., Pehar M. (2008). Mitochondrial dysfunction in SOD1G93A-bearing astrocytes promotes motor neuron degeneration: prevention by mitochondrial-targeted antioxidants. *The Journal of Neuroscience*.

[265] Miquel E., Cassina A., Martínez-Palma L. (2014). Neuroprotective effects of the mitochondria-targeted antioxidant MitoQ in a model of inherited amyotrophic lateral sclerosis. *Free Radical Biology & Medicine*.

[266] Gane E. J., Weilert F., Orr D. W. (2010). The mitochondria-targeted anti-oxidant mitoquinone decreases liver damage in a phase II study of hepatitis C patients. *Liver International*.

[267] Zielonka J., Joseph J., Sikora A. (2017). Mitochondria-targeted triphenylphosphonium-based compounds: syntheses, mechanisms of action, and therapeutic and diagnostic applications. *Chemical Reviews*.

[268] Pehar M., Vargas M. R., Robinson K. M. (2007). Mitochondrial superoxide production and nuclear factor erythroid 2-related factor 2 activation in p75 neurotrophin receptor-induced motor neuron apoptosis. *The Journal of Neuroscience*.

[269] Petri S., Kiaei M., Damiano M. (2006). Cell-permeable peptide antioxidants as a novel therapeutic approach in a mouse model of amyotrophic lateral sclerosis. *Journal of Neurochemistry*.

[270] Zhao K., Zhao G. M., Wu D. (2004). Cell-permeable peptide antioxidants targeted to inner mitochondrial membrane inhibit mitochondrial swelling, oxidative cell death, and reperfusion injury. *The Journal of Biological Chemistry*.

[271] Zhao K., Luo G., Giannelli S., Szeto H. H. (2005). Mitochondria-targeted peptide prevents mitochondrial depolarization and apoptosis induced by tert-butyl hydroperoxide in neuronal cell lines. *Biochemical Pharmacology*.

[272] Scott S., Kranz J. E., Cole J. (2009). Design, power, and interpretation of studies in the standard murine model of ALS. *Amyotrophic Lateral Sclerosis*.

[273] Ludolph A. C., Bendotti C., Blaugrund E. (2010). Guidelines for preclinical animal research in ALS/MND: a consensus meeting. *Amyotrophic Lateral Sclerosis*.

[274] Benatar M. (2007). Lost in translation: treatment trials in the SOD1 mouse and in human ALS. *Neurobiology of Disease*.

[275] Pollari E., Goldsteins G., Bart G. Ã.¨., Koistinaho J., Giniatullin R. (2014). The role of oxidative stress in degeneration of the neuromuscular junction in amyotrophic lateral sclerosis. *Frontiers in Cellular Neuroscience*.

[276] Morrice J. R., Gregory-Evans C. Y., Shaw C. A. (2018). Animal models of amyotrophic lateral sclerosis: a comparison of model validity. *Neural Regeneration Research*.

[277] Lutz C. (2018). Mouse models of ALS: past, present and future. *Brain Research*.

[278] Bossolasco P., Sassone F., Gumina V., Peverelli S., Garzo M., Silani V. (2018). Motor neuron differentiation of iPSCs obtained from peripheral blood of a mutant TARDBP ALS patient. *Stem Cell Research*.

[279] Russo F. B., Cugola F. R., Fernandes I. R., Pignatari G. C., Beltrão-Braga P. C. (2015). Induced pluripotent stem cells for modeling neurological disorders. *World Journal of Transplantation*.

